# Lysosomal TPC2 channels disrupt Ca^2+^ entry and dopaminergic function in models of LRRK2-Parkinson’s disease

**DOI:** 10.1083/jcb.202412055

**Published:** 2025-04-25

**Authors:** Martina Gregori, Gustavo J.S. Pereira, Robert Allen, Nicholas West, Kai-Yin Chau, Xinjiang Cai, Matthew P. Bostock, Stephen R. Bolsover, Marco Keller, Chiao-Yin Lee, Si Hang Lei, Kirsten Harvey, Franz Bracher, Christian Grimm, Gaiti Hasan, Matthew E. Gegg, Anthony H.V. Schapira, Sean T. Sweeney, Sandip Patel

**Affiliations:** 1 Department of Cell and Developmental Biology, University College London, London, UK; 2 Department of Pharmacology, Federal University of São Paulo (UNIFESP), São Paulo, Brazil; 3 Department of Biology, University of York, York, UK; 4 Department of Clinical and Movement Neurosciences, UCL Queen Square Institute of Neurology, University College London, London, UK; 5 Division of Cardiology, David Geffen School of Medicine at UCLA, Los Angeles, CA, USA; 6 Centre for Developmental Neurobiology and MRC Centre for Neurodevelopmental Disorders, King’s College London, London, UK; 7 Department of Pharmacy-Center for Drug Research, Ludwig-Maximilian University, Munich, Germany; 8 Department of Pharmacology, UCL School of Pharmacy, London, UK; 9 Walther Straub Institute of Pharmacology and Toxicology, Faculty of Medicine, Ludwig-Maximilian University, Munich, Germany; 10 Immunology, Infection and Pandemic Research IIP, Fraunhofer Institute for Translational Medicine and Pharmacology ITMP, Frankfurt, Germany; 11 National Centre for Biological Sciences, Tata Institute of Fundamental Research, Bangalore, India

## Abstract

Parkinson’s disease results from degeneration of dopaminergic neurons in the midbrain, but the underlying mechanisms are unclear. Here, we identify novel crosstalk between depolarization-induced entry of Ca^2+^ and lysosomal cation release in maintaining dopaminergic neuronal function. The common disease-causing G2019S mutation in LRRK2 selectively exaggerated Ca^2+^ entry in vitro. Chemical and molecular strategies inhibiting the lysosomal ion channel TPC2 reversed this. Using *Drosophila*, which lack TPCs, we show that the expression of human TPC2 phenocopied LRRK2 G2019S in perturbing dopaminergic-dependent vision and movement in vivo. Mechanistically, dysfunction required an intact pore, correct subcellular targeting and Rab interactivity of TPC2. Reducing Ca^2+^ permeability with a novel biased TPC2 agonist corrected deviant Ca^2+^ entry and behavioral defects. Thus, both inhibition and select activation of TPC2 are beneficial. Functional coupling between lysosomal cation release and Ca^2+^ influx emerges as a potential druggable node in Parkinson’s disease.

## Teaser

Death of dopaminergic neurons in the brain is a hallmark of Parkinson’s disease. Gregori and colleagues show that deviant calcium signals in familial disease models caused by mutations in LRRK2 can be reversed by targeting lysosomal TPC2 channels and that TPC2 mimics disease effects in vivo.

## Introduction

Parkinson’s disease (PD) is the most common movement disorder and the second most common neurodegenerative disease after Alzheimer’s disease (Balestrino and Schapira, 2020). The dominant early motor features of PD result from the selective degeneration of dopaminergic neurons within the substantia nigra pars compacta. Additional nonmotor symptoms such as cognitive dysfunction are increasingly common as the disease progresses (Schapira et al., 2017) with others such as visual changes even manifesting during the presymptomatic prodrome to PD (McNeill et al., 2013). Mutations in the leucine-rich repeat kinase 2 (*LRRK2*) gene are a common cause of familial PD, and patients carrying them present with a phenotype clinically and pathologically indistinguishable from idiopathic disease (Di Fonzo et al., 2006; Paisán-Ruíz et al., 2004; Zimprich et al., 2004). Pathogenic LRRK2 drives deficits in neuronal morphology (MacLeod et al., 2006) and synaptic transmission (Afsari et al., 2014; Arranz et al., 2015; Matta et al., 2012; Piccoli et al., 2014) including dopamine and glutamate release (Liu et al., 2015; Volta et al., 2017). LRRK2 is a large, multidomain signaling protein with the most common G2019S PD-linked mutation falling within the kinase domain (West et al., 2005). A subset of the Rab family of trafficking GTPases are bona fide LRRK2 substrates (Steger et al., 2016), but few others have been confirmed. Thus, the function of LRRK2 remains enigmatic with a clear need to delineate downstream targets as alternative ways to combat LRRK2 defects and PD more generally.

Ca^2+^ is key for neuronal homeostasis (Berridge, 1998). Oscillatory Ca^2+^ influx into the cytoplasm is associated intimately with spontaneous firing of dopaminergic neurons in the substantia nigra (Guzman et al., 2009; Nedergaard et al., 1993). This flux through voltage-gated Ca^2+^ channels has attracted therapeutic attention not least due to epidemiological evidence linking Ca^2+^ channel blocker use to reduced risk of PD (Chan et al., 2007; Ritz et al., 2010). Downstream of Ca^2+^ entry, excessive Ca^2+^ uptake by mitochondria potentially links Ca^2+^ homeostasis disruption to the established role of mitochondria and oxidative stress in the disease (Guzman et al., 2010; Surmeier et al., 2017). In addition to mitochondrial dysfunction, growing evidence links lysosomal dysfunction to PD (Dehay et al., 2013; Platt, 2014; Smith et al., 2022). Genetics have identified mutations in the *GBA1* gene, which encodes a lysosomal hydrolase (Sidransky et al., 2009), as the most common risk factor for PD. Additionally, large-scale sequencing efforts of sporadic PD patients identified an excessive burden of variants in genes that cause lysosomal storage diseases (Robak et al., 2017). LRRK2 localizes to the endo-lysosomal system (Alegre-Abarrategui et al., 2009; Biskup et al., 2006; Dodson et al., 2012; Schapansky et al., 2018) particularly upon stress (Eguchi et al., 2018) and has been heavily implicated in endo-lysosomal trafficking (Madureira et al., 2020; Manzoni and Lewis, 2013), morphology, and repair (Bonet-Ponce et al., 2020; Herbst et al., 2020). There is much evidence now that the endo-lysosomal system serves as a functionally relevant Ca^2+^ store (Galione and Muallem, 2023; Patel and Muallem, 2011). But the potential role of lysosomal Ca^2+^ signaling dysfunction in PD and neurodegeneration more generally is underexplored (Patel, 2016).

Here, we used human and fly models, a number of in vitro and in vivo assays including Ca^2+^ imaging together with novel pharmacological manipulation to identify a role for the lysosomal cation channel two-pore channel-2 (TPC2) in Ca^2+^ dysfunction and dopaminergic deficiency in LRRK2 PD. TPC2, together with TPC1, is an ancient member of the voltage-gated ion channel superfamily regulated by the signaling molecules, NAADP and PI(3,5P)_2_ (Brailoiu et al., 2009a; Calcraft et al., 2009; Wang et al., 2012). TPC2 has the highly unusual ability to function as a Ca^2+^ or Na^+^ channel depending on the activating stimulus (Gerndt et al., 2020; Yuan et al., 2022). It is a Rab effector (Abrahamian et al., 2024; Lin-Moshier et al., 2014), regulates numerous cellular processes including many aspects of membrane traffic (Grimm et al., 2014; Kilpatrick et al., 2017; Sakurai et al., 2015), and has previously been shown to mediate autophagic dysfunction (Gómez-Suaga et al., 2012), lysosomal morphology defects (Hockey et al., 2015), and aberrant activation of the lysosomal transcription factor, TFEB (Nabar et al., 2022), in response to mutant LRRK2 in nonexcitable cells (Hockey et al., 2015). Here, we report neuronal Ca^2+^ entry defects in response to pathogenic LRRK2 in vitro, a key role of TPC2 in mediating these defects and TPC2-dependent disruptions in dopaminergic circuits that drive vision and movement in vivo. We further exploit the malleable ion selectivity of TPC2 to pharmacologically restore Ca^2+^ homeostasis and behavioral well-being. These data suggest a novel strategy for slowing PD.

## Results

### Pathogenic LRRK2 deregulates depolarization-induced Ca^2+^ entry

**Figure 1.**
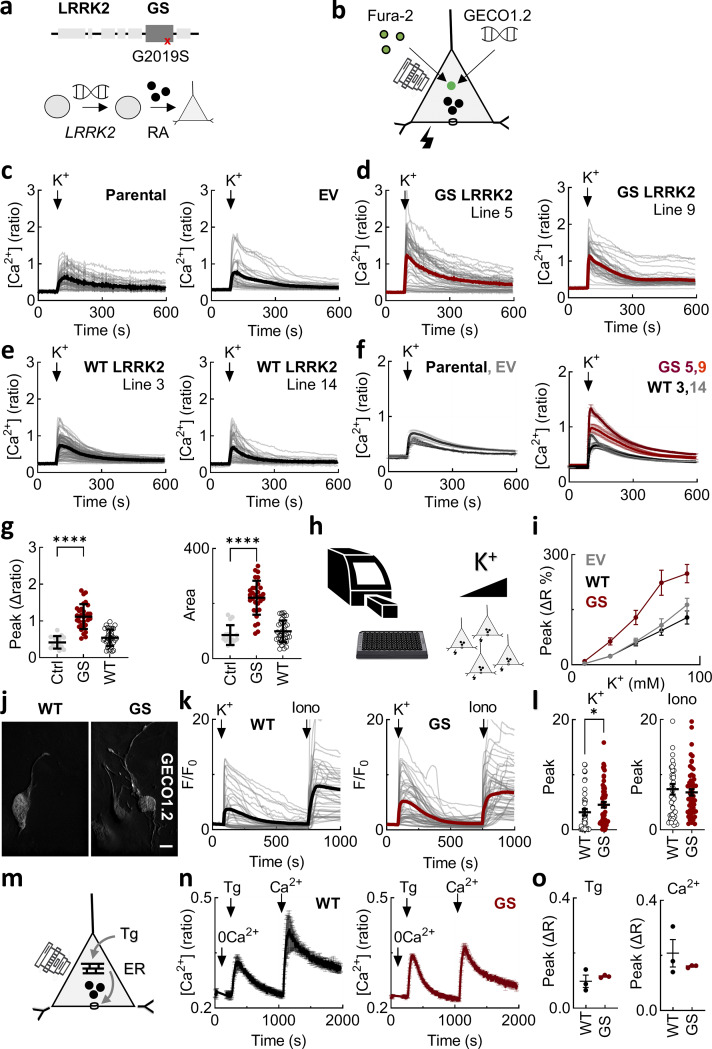
**Pathogenic LRRK2 deregulates depolarization-induced Ca** ^**2+**^ **entry. (a)** Schematic of LRRK2 showing the position of the G2019S mutation (GS) linked to PD. Human SH-SY5Y cell lines stably expressing LRRK2 were generated and then differentiated with RA for analyses. **(b)** Schematic of the imaging technique used to monitor changes in cytosolic Ca^2+^ through Ca^2+^ channels in the cell membrane in response to K^+^ depolarization using chemical (Fura-2) or genetically encoded (GECO1.2) fluorescent indicators. **(c–e)** Exemplar Ca^2+^ signals recorded from cells loaded with Fura-2 from the indicated cell line in response to 50 mM K^+^. Gray lines are responses from individual cells. The thick lines are the population average. **(f)** Ca^2+^ signals from multiple population averages (mean ± SEM) of the indicated line. *n* = 3 (parental), *n* = 12 (EV), *n* = 24 (GS 5), *n* = 7 (GS 9), *n* = 18 (WT 3), *n* = 18 (WT 14), where n refers to the number of independent biological replicates. **(g)** Summary data (mean ± SEM) quantifying the peak change and the area under the curve for the Ca^2+^ signals in the indicated line. Data were amalgamated for the control lines (parental and EV (*n* = 15)) and lines expressing wild-type LRRK2 (*n* = 36) and LRRK2 G2019S (*n* = 31). Each point represents the mean response from a cell population. ****P < 0.0001 (one-way ANOVA, Tukey’s test). **(h)** Schematic of the method used to record changes in cytosolic Ca^2^ in cell populations through Ca^2+^ channels in the cell membrane in response to increasing concentrations of K^+^. **(i)** Summary data (mean ± SEM) quantifying the peak change in the Ca^2+^ signals from the indicated line and K^+^ concentration. *n* = 10 (EV), *n* = 13 (GS), *n* = 13 (WT), where n refers to the number of independent biological replicates. **(j)** Confocal micrographs of cells expressing GECO1.2. Scale bar: 10 µm. **(k)** Exemplar Ca^2+^ signals recorded from cells expressing GECO1.2 from the indicated cell line. Cells were stimulated with K^+^ (50 mM) and ionomycin (10 µM) toward the end of the recording. Gray lines are responses from individual cells. The thick line is the population average. **(l)** Summary data (mean ± SEM) quantifying the peak change in the Ca^2+^ signals from the indicated line. Each point represents the response from an individual transfected cell. *n* = 44 (WT), *n* = 66 (GS), where n refers to the number of cells from three independent transfections. *P < 0.05 (Mann–Whitney test). **(m)** Schematic depicting entry of Ca^2+^ through store-operated Ca^2+^ channels in the cell membrane in response to depletion of ER Ca^2+^ stores with Tg. **(n)** Ca^2+^ signals (mean ± SEM, *n* = 3 independent biological replicates) in response to thapsigargin (1 µM). Cells were loaded with Fura-2 and stimulated in the absence of external Ca^2+^, and then, external Ca^2+^ was added back as indicated. **(o)** Summary data (mean ± SEM, *n* = 3 independent biological replicates) quantifying the peak change in the Ca^2+^ signals from the indicated line. Each point represents the mean response from a cell population. ns, nonsignificant (unpaired *t* test). RA, retinoic acid; Tg, thapsigargin; EV, empty vector.

**Figure S1.**
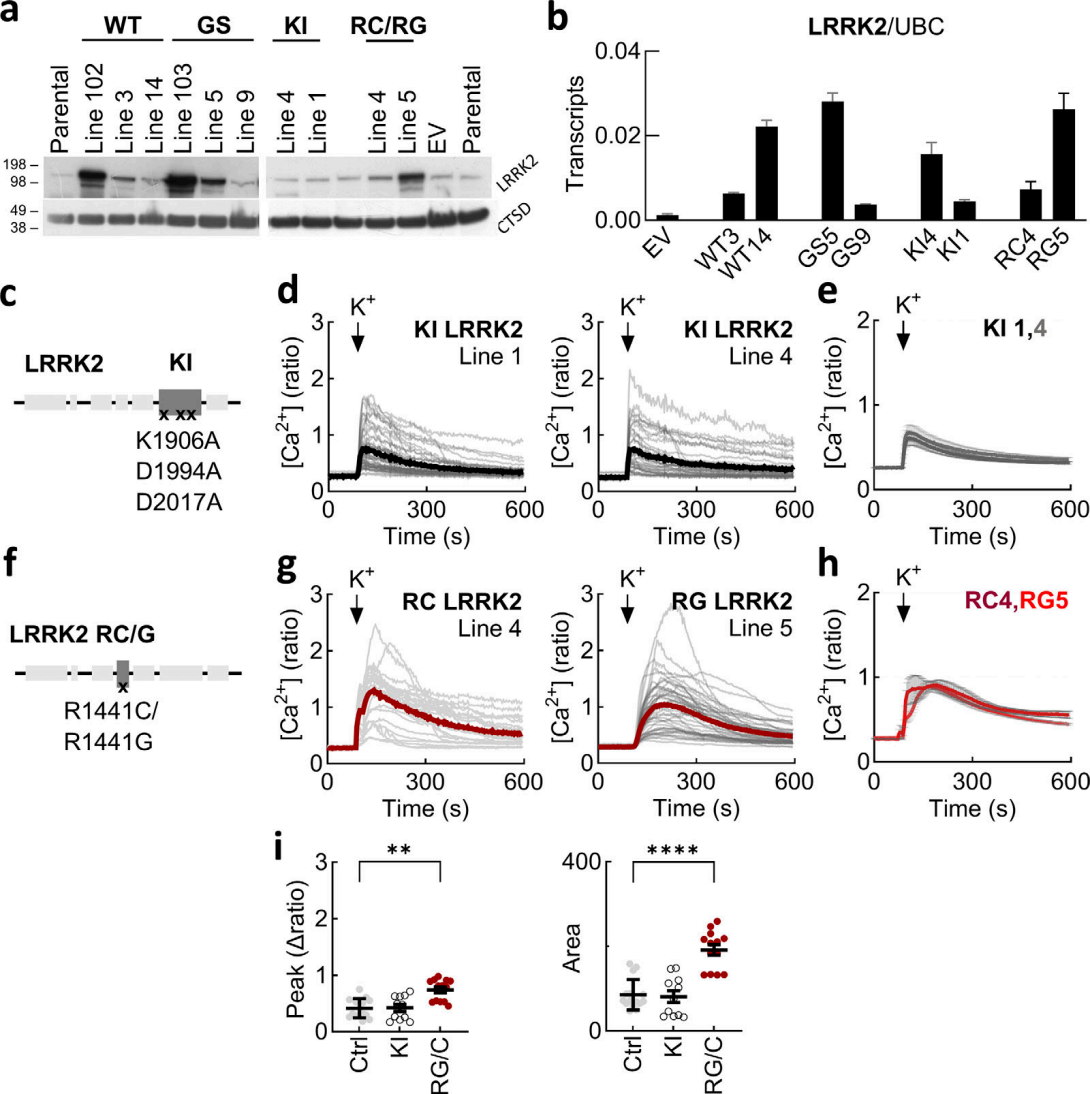
**Validation of LRRK2-expressing SH-SY5Y cell lines and effects of ROC/COR and kinase-inactivating mutations on cytosolic Ca** ^**2+**^ **. (a)** Western blot analyses using an antibody to LRRK2 in the parental SH-SY5Y cell line and cell lines expressing EV, wild-type LRRK2 (WT), LRRK2 G2019S (GS), KI LRRK2 (KI), LRRK2 R1441C (RC), and LRRK2 R1441G (RG). Expression of wild-type and G2019S LRRK2 in published lines (102 and 103, respectively)^52^ was processed in parallel. Blots were reprobed with an antibody to cathepsin D (CTSD). **(b)** Quantitative PCR analysis of LRRK2 in the various cell lines used in this study. Data (mean ± SD, *n* = 3 technical replicates) are normalized to the expression of ubiquitin. **(c)** Schematic of LRRK2 showing the position of the three mutations introduced to generate the KI construct and the two individual ROC/COR mutations (RG/RC). **(d)** Exemplar Ca^2+^ signals recorded from cells loaded with Fura-2 from the lines expressing KI LRRK2. Gray lines are responses from individual cells. The thick lines are the population average. **(e)** Ca^2+^ signals from multiple population averages (mean ± SEM). *n* = 6 (KI 1), *n* = 5 (KI 4), where n refers to the number of independent biological replicates. **(f)** Schematic of LRRK2 showing the position of the two individual ROC/COR mutations (RG/RC). **(g)** Exemplar Ca^2+^ signals recorded from cells loaded with Fura-2 from the lines expressing the ROC/COR LRRK2 mutants. Gray lines are responses from individual cells. The thick lines are the population average. **(h)** Ca^2+^ signals from multiple population averages (mean ± SEM). *n* = 5 (RC 4), *n* = 8 (RG 5), where n refers to the number of independent biological replicates. **(i)** Summary data quantifying the peak change and the area under the curve for the Ca^2+^ signals. Data are presented as the mean ± SEM and amalgamated for both lines expressing KI LRRK2 and the ROC/COR mutants where each point is an independent biological replicate. Data are compared with amalgamated control lines (parental and empty vector). **P < 0.01 (Kruskal–Wallis, Dunn’s test), ****P < 0.0001 (one-way ANOVA, Tukey’s test). WT, wild type; EV, empty vector; KI, kinase inactive. Source data are available for this figure: SourceData FS1.

To examine the effects of pathogenic LRRK2 on neuronal Ca^2+^ homeostasis, we generated dopaminergic SH-SY5Y cell lines stably expressing either wild-type LRRK2 or the common PD-causing G2019S mutant (Fig. 1 a). We analyzed expression by western blotting (Fig. S1 a) and quantitative PCR (Fig. S1 b) and selected two clones of each genotype with differing expression levels for our analyses. LRRK2 expression was lower than in cell lines established previously (Papkovskaia et al., 2012) and much nearer endogenous levels (Fig. S1 a), thereby providing a more physiological readout of LRRK2 action.

To measure neuronal activity, we monitored cytosolic Ca^2+^ levels in individual differentiated cells loaded with the Ca^2+^ indicator Fura-2 (Fig. 1 b). Cells were depolarized with 50 mM K^+^ to stimulate Ca^2+^ entry from outside of the cell via voltage-gated Ca^2+^ channels. As shown in Fig. 1 c, depolarization induced Ca^2+^ transients in the parental line. The responses were similar in cells expressing the empty vector. In marked contrast, Ca^2+^ responses in the two independent cell lines expressing LRRK2 G2019S were increased (Fig. 1 d). To assess the specificity of this effect, we took two approaches. In the first approach, we measured depolarization-induced responses in cells expressing wild-type LRRK2. Ca^2+^ entry was reduced relative to the mutant lines in both of the lines tested resulting in signals comparable to the cells lacking exogenous LRRK2 (Fig. 1 e). In the second approach, we analyzed cell lines expressing kinase-inactive LRRK2 (Fig. S1 c). Again, Ca^2+^ entry was reduced in both of the lines tested relative to the LRRK2 G2019S lines and similar to the control cells (Fig. S1, d and e). We also analyzed lines expressing the R1441C or R1441G mutations within the ROC/COR domain of LRRK2 (Fig. S1 f). These lines showed exaggerated Ca^2+^ signals although the effects were less pronounced than the G2019S mutation (Fig. S1, g and h). Summary data quantifying both the peak response and the area under the curve across lines are shown in Fig. 1, f and g; and Fig. S1 i.

We used automated plate reading coupled with microfluidics to obtain full concentration–effect relationships for extracellular K^+^ and intracellular Ca^2+^ (Fig. 1 h). K^+^ evoked Ca^2+^ responses in a concentration-dependent manner in cells expressing the empty vector (Fig. 1 i). Ca^2+^ responses in cells expressing LRRK2 G2019S were increased at all K^+^ concentrations relative to the empty vector. In contrast, Ca^2+^ responses in cells expressing wild-type LRRK2 were not (Fig. 1 i).

We also performed experiments using cells expressing the genetically encoded Ca^2+^ indicator, GECO1.2 (Fig. 1 b). This was to mitigate against any off-target effects of the chemical indicator for Ca^2+^ on membrane potential (Smith et al., 2018). As shown in Fig. 1 j, GECO1.2 expression was readily detectable in wild-type and LRRK2 G2019S–expressing cells. Robust depolarization-evoked Ca^2+^ responses were recorded, and similar to the responses in cells loaded with Fura-2, the responses were larger in the LRRK2 G2019S line (Fig. 1 k). In contrast, Ca^2+^ signals evoked following subsequent stimulation of the same cells with the Ca^2+^ ionophore ionomycin were similar (Fig. 1, k and l), attesting to specificity.

To further test the specificity of the G2019S effect, we compared store-operated Ca^2+^ entry in the wild-type and mutant cells. Endoplasmic reticulum Ca^2+^ store depletion was induced by treating cells with the SERCA inhibitor thapsigargin in the absence of external Ca^2+^ (Fig. 1 m) As shown in Fig. 1 n, thapsigargin evoked a Ca^2+^ signal consistent with leak of Ca^2+^ from the endoplasmic reticulum. Subsequent addition of external Ca^2+^ evoked Ca^2+^ entry. Neither the initial response to thapsigargin nor the subsequent one to Ca^2+^ was different in the LRRK2 G2019S cells compared with the controls (Fig. 1, n and o), again attesting to specificity.

In sum, we show depolarization-induced Ca^2+^ entry is selectively exaggerated by pathogenic LRRK2.

### LRRK2-induced Ca^2+^ entry defects are reversed by targeting lysosomal TPC2

**Figure 2.**
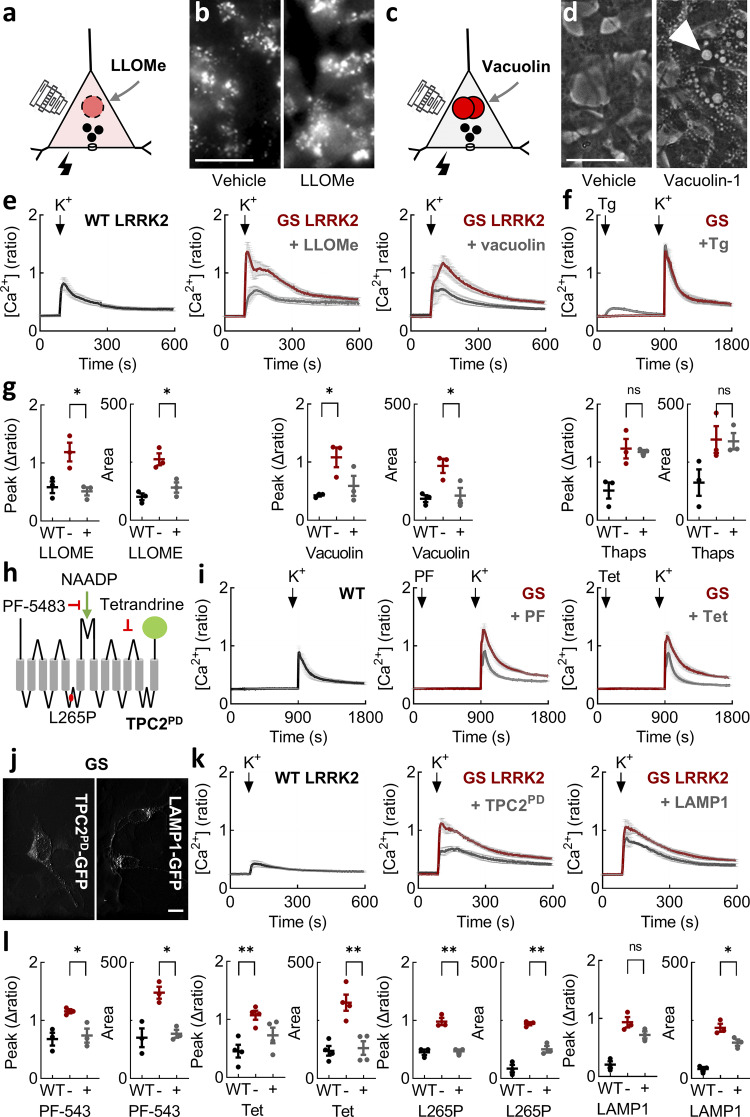
**LRRK2-induced Ca** ^**2+**^ **entry defects are reversed by targeting lysosomal TPC2. (a)** Schematic depicting permeabilization of lysosomes by LLOMe. **(b)** Epifluorescence micrographs of the indicated cell line loaded with rhodamine B–dextran and treated with vehicle (DMSO) or LLOMe (1 mM) for 1 h. Scale bar: 100 µm. **(c)** Schematic depicting fusion of lysosomes by vacuolin-1. **(d)** Transmitted light micrographs of the indicated cell line treated with vehicle (DMSO) or vacuolin-1 (1 µM) overnight. Arrows highlight the presence of vacuoles. Scale bar: 100 µm. **(e)** Effect of LLOMe and vacuolin-1 on depolarization-evoked Ca^2+^ signals in the indicated cell type (mean ± SEM, *n* = 3 independent biological replicates). **(f)** Effect of thapsigargin on depolarization-evoked Ca^2+^ signals in cells expressing LRRK2 G2019S (mean ± SEM, *n* = 3 independent biological replicates). **(g)** Summary data (mean ± SEM, *n* = 3 independent biological replicates) from e and f quantifying the peak change in the Ca^2+^ signals from the indicated line and treatment. Each point represents the mean response from a cell population. *P < 0.05 (one-way ANOVA, Tukey’s test). **(h)** Schematic of TPC2 depicting blockade of NAADP activation by PF-543 and the channel pore by Tet or mutation of Leu265. **(i)** Effect of PF-5483 and Tet on depolarization-evoked Ca^2+^ signals in the indicated cell type (mean ± SEM, *n* = 3–4 independent biological replicates). **(j)** Confocal micrographs of cells expressing TPC2^PD^-GFP or LAMP1-GFP. Scale bar: 10 µm. **(k)** Effect of TPC2^PD^ and LAMP 1 on depolarization-evoked Ca^2+^ signals in the indicated cell type (mean ± SEM, *n* = 3 independent biological replicates). **(l)** Summary data (mean ± SEM, *n* = 3–4 independent biological replicates) from i and k quantifying the peak change in the Ca^2+^ signals and area under the curve from the indicated line and treatment. Each point represents the mean response from a cell population. *P < 0.05, **P < 0.01 (one-way ANOVA, Tukey’s test). Tet, tetrandrine.

**Figure S2.**
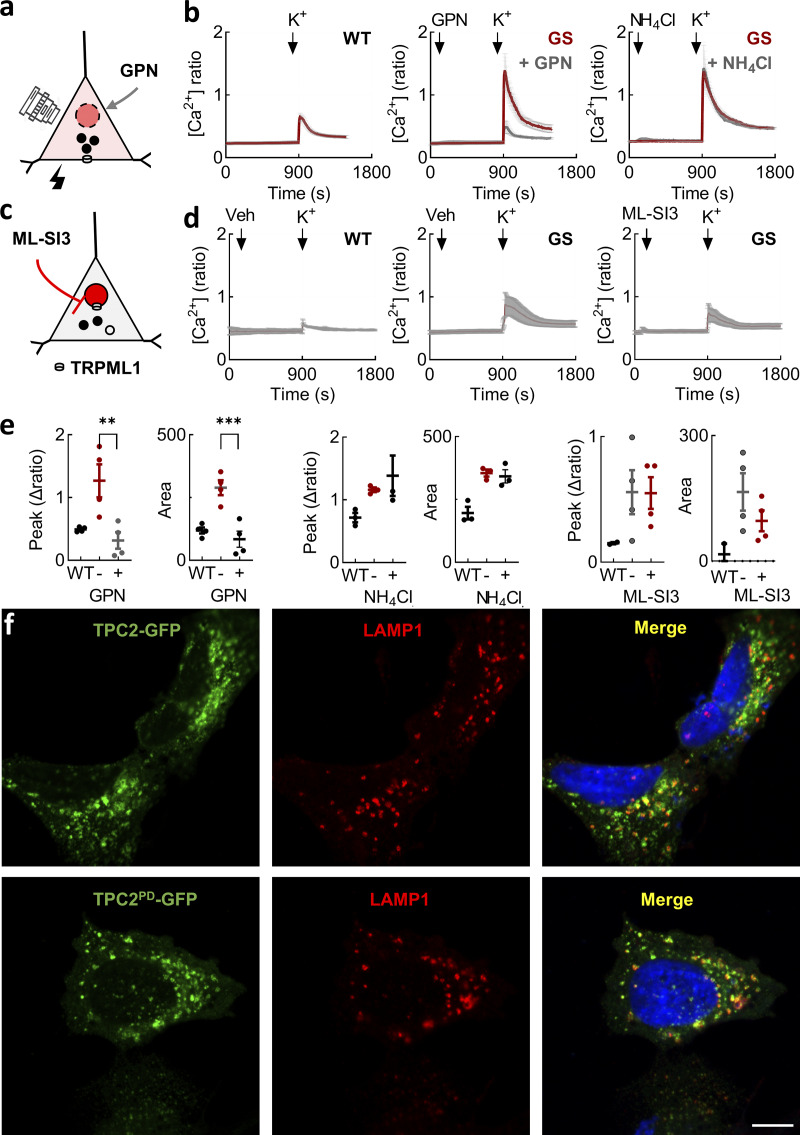
**Effects of disrupting lysosomes on cytosolic Ca** ^**2+**^ **in SH-SY5Y cells. (a)** Schematic depicting permeabilization of lysosomes by GPN. **(b)** Effect of GPN (50 µM) and NH_4_Cl (5 mM) on depolarization-evoked Ca^2+^ signals in the indicated cell type (mean ± SEM, *n* = 3–4 independent biological replicates). **(c)** Schematic depicting inhibition of the lysosomal ion channel TRPML1 by the small molecule inhibitor, ML-SI3. **(d)** Effect of ML-SI3 (10 µM) on depolarization-evoked Ca^2+^ signals in the indicated cell type (mean ± SEM, *n* = 2–4 independent biological replicates). **(e)** Summary data (mean ± SEM from two to four independent biological replicates) quantifying the peak change in the Ca^2+^ signals and the area under the curve from the indicated cell line and treatment. Each point represents the mean response from a cell population. ns, nonsignificant, **P < 0.01, ***P < 0.001 (one-way ANOVA, Tukey’s test). **(f)** Confocal micrographs of SH-SY5Y cells expressing GFP-tagged TPC2 and TPC2^PD^ expressed in SH-SY5Y cells (green). Cells were counterstained with an antibody to LAMP-1 (red). Merged images are shown to the right. Scale bar: 7 µm.

Lysosomes are increasingly implicated in the actions of LRRK2 and PD more generally (Dehay et al., 2013; Platt, 2014; Smith et al., 2022). To probe the role of lysosomes in Ca^2+^ dysfunction mediated by LRRK2 G2019S, we began by disrupting lysosome integrity. We did this in two ways (Fig. 2 a). In the first, we treated cells with the lysosome-permeabilizing agent LLOMe (Fig. 2 a). As shown in Fig. 2 b, LLOMe changed the subcellular distribution of endocytosed dextran from a punctate one to a more diffuse one consistent with a mild permeabilizing effect. This treatment was sufficient to reverse the potentiating effects of the G2019S mutant on depolarization-induced Ca^2+^ entry (Fig. 2 e). Essentially, similar results were obtained with a structurally distinct cathepsin C substrate, GPN (Fig. S2, a and b). This was not due to changes in lysosomal pH (Atakpa et al., 2019) because NH_4_Cl did not affect Ca^2+^ entry in LRRK2 G2019S–expressing cells (Fig. S2, a and b). In the second, we treated cells with vacuolin-1, which promotes endo-lysosomal fusion (Fig. 2 c). Consistent with this action, vacuolin-1 induced the appearance of large cytoplasmic vesicles (Fig. 2 d). As with LLOMe, vacuolin-1 also reversed Ca^2+^ defects induced by the G2019S mutant (Fig. 2 e). To probe the role of the endoplasmic reticulum in LRRK2 G2019S action, we depolarized cells following depletion of endoplasmic reticulum Ca^2+^ stores with thapsigargin. Thapsigargin had little effect on depolarization-induced Ca^2+^ entry (Fig. 2 f). Taken together, these data summarized in Fig. 2 g show that interfering with lysosomal integrity reverses Ca^2+^ defects evoked by LRRK2 G2019S.

To probe the mechanisms underlying LRRK2-mediated Ca^2+^ defects, we considered a role for TPC2 because TPC2 is a lysosomal ion channel and inhibiting it reverses trafficking defects in LRRK2 G2019S patient fibroblasts (Hockey et al., 2015). Ca^2+^ release through TPC2 is activated indirectly by the second messenger NAADP (Fig. 2 h) (Marchant et al., 2022). We targeted this axis in two ways. In the first pharmacological approach, we used the recently described NAADP antagonist PF-543 (Gunaratne et al., 2018). As shown in Fig. 2 i, PF543 reversed the effects of LRRK2 G2019S. We also targeted the channel more directly with the TPC2 blocker tetrandrine (Fig. 2 g). Tetrandrine reversed the effects of LRRK2 G2019S (Fig. 2 i). In contrast, chemical inhibition of lysosomal TRP mucolipin channels with ML-SI3 had little effect (Fig. S2, c–e). In the second approach, we expressed TPC2 mutated at Leu265 within the pore region (Fig. 2 h) (Brailoiu et al., 2010b). This construct (TPC2^PD^, for pore-dead) acts in a dominant negative way to block NAADP action. GFP-tagged TPC2^PD^ was expressed on punctate structures similar to LAMP1-GFP (Fig. 2 j) and colocalized with endogenous LAMP1 similar to wild-type TPC2 (Fig. S2 f). As shown in Fig. 2 k, depolarization-evoked Ca^2+^ entry in LRRK2 G2019S–expressing cells was reduced in cells expressing TPC2^PD^ relative to neighboring untransfected cells. In contrast, the overexpression of LAMP1 had little effect. Pooled data quantifying the effects of PF-543, tetrandrine, and TPC2^PD^ on Ca^2+^ entry are shown in Fig. 2 l.

In sum, both chemical and molecular strategies inhibiting TPC2 normalized defective Ca^2+^ entry caused by pathogenic LRRK2.

### TPC2 activation regulates Ca^2+^ entry in an agonist-selective manner

**Figure 3.**
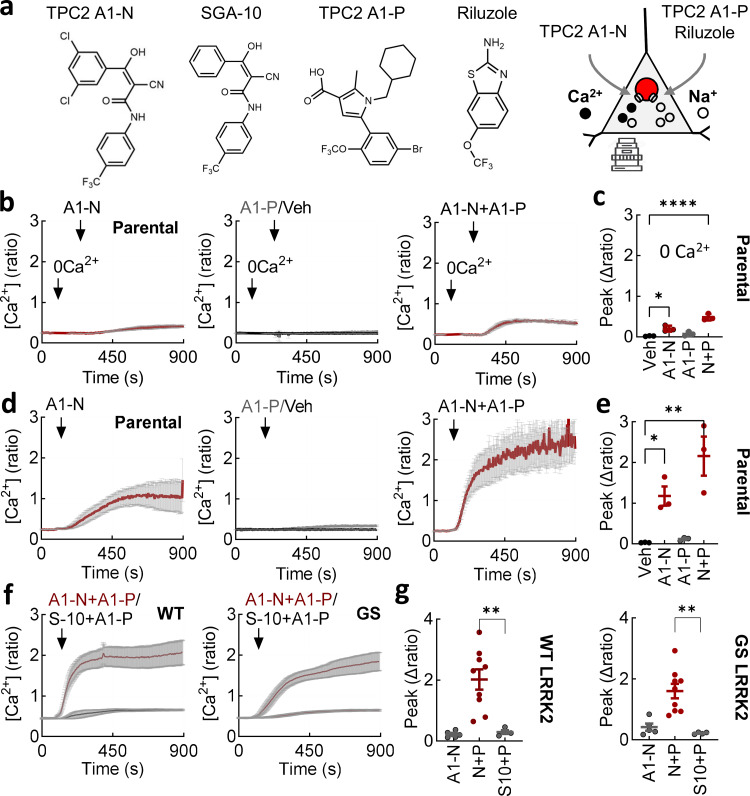
**TPC2 activation regulates Ca** ^**2+**^ **entry in an agonist-selective manner. (a)** Chemical structures of the TPC2 agonists TPC2-A1-N, its inactive analog SGA-10, TPC2-A1-P, and riluzole. Schematic depicts activation of TPC2-mediated Ca^2+^ flux by TPC2-A1-N and TPC2-mediated Na^+^ flux by TPC2-A1-P and riluzole. **(b)** Ca^2+^ signals (mean ± SEM, *n* = 3 independent biological replicates) in response to TPC2-A1-N (30 µM), TPC2-A1-P (30 µM), and a combination of the two. Parental cells were loaded with Fura-2 and stimulated in the absence of external Ca^2+^ using DMSO as a vehicle (Veh.) control. **(c)** Summary data (mean ± SEM, *n* = 3 independent biological replicates) quantifying the peak change in the Ca^2+^ signals. Each point represents the mean response from a cell population. *P < 0.05, ****P < 0.0001 (one-way ANOVA, Dunnett’s test). **(d)** Ca^2+^ signals (mean ± SEM, *n* = 3 independent biological replicates) in response to TPC2-A1-N (30 µM), TPC2-A1-P (30 µM), and a combination of the two. Parental cells were loaded with Fura-2 and stimulated in the presence of external Ca^2+^ using DMSO as a vehicle (Veh.) control. **(e)** Summary data (mean ± SEM, *n* = 3 independent biological replicates) quantifying the peak change in the Ca^2+^ signals. Each point represents the mean response from a cell population. *P < 0.05, **P < 0.01 (one-way ANOVA, Dunnett’s test). **(f)** Ca^2+^ signals (mean ± SEM, *n* = 4–9 independent biological replicates) in response to TPC2-A1-N (30 µM) or SGA-10 (30 µM) in combination with TPC2-A1-P (30 µM). Cells expressing wild-type or G2019S LRRK2 were loaded with Fura-2 and stimulated in the presence of external Ca^2+^. **(g)** Summary data (mean ± SEM, *n* = 4–9 independent biological replicates) quantifying the peak change in the Ca^2+^ signals in the indicated treatment and line. Each point represents the mean response from a cell population. **P < 0.01 (one-way ANOVA, Tukey’s test).

To investigate the link between lysosomal Ca^2+^ release and Ca^2+^ entry, we leveraged the availability of recently described cell-permeable TPC2 agonists that bias the channel to either a Ca^2+^-permeable, NAADP activated–like state or a more Na^+^-selective, PI(3,5)P_2_ activated–like state (Fig. 3 a) (Gerndt et al., 2020).

As shown in Fig. 3 b, the NAADP-mimetic TPC2-A1-N evoked a modest but detectable Ca^2+^ signal in parental SH-SY5Y cells. These experiments were performed in the absence of external Ca^2+^ to isolate Ca^2+^ release. TPC2 is also activated by PI(3,5)P_2_, but in stark contrast to NAADP, it evokes largely Na^+^-selective currents (Gerndt et al., 2020). This “switch” can be mimicked by TPC2-A1-P (Fig. 3 a). TPC2-A1-P had little effect on cytosolic Ca^2+^ consistent with signaling through Na^+^ (Fig. 3 b). Costimulation of TPC2 with its agonists, however, caused a significantly increased Ca^2+^ response (Fig. 3, b and c), indicating that the recently reported synergistic activation of TPC2 in nonexcitable cells (Yuan et al., 2022) is a feature of excitable cells too.

**Figure S3.**
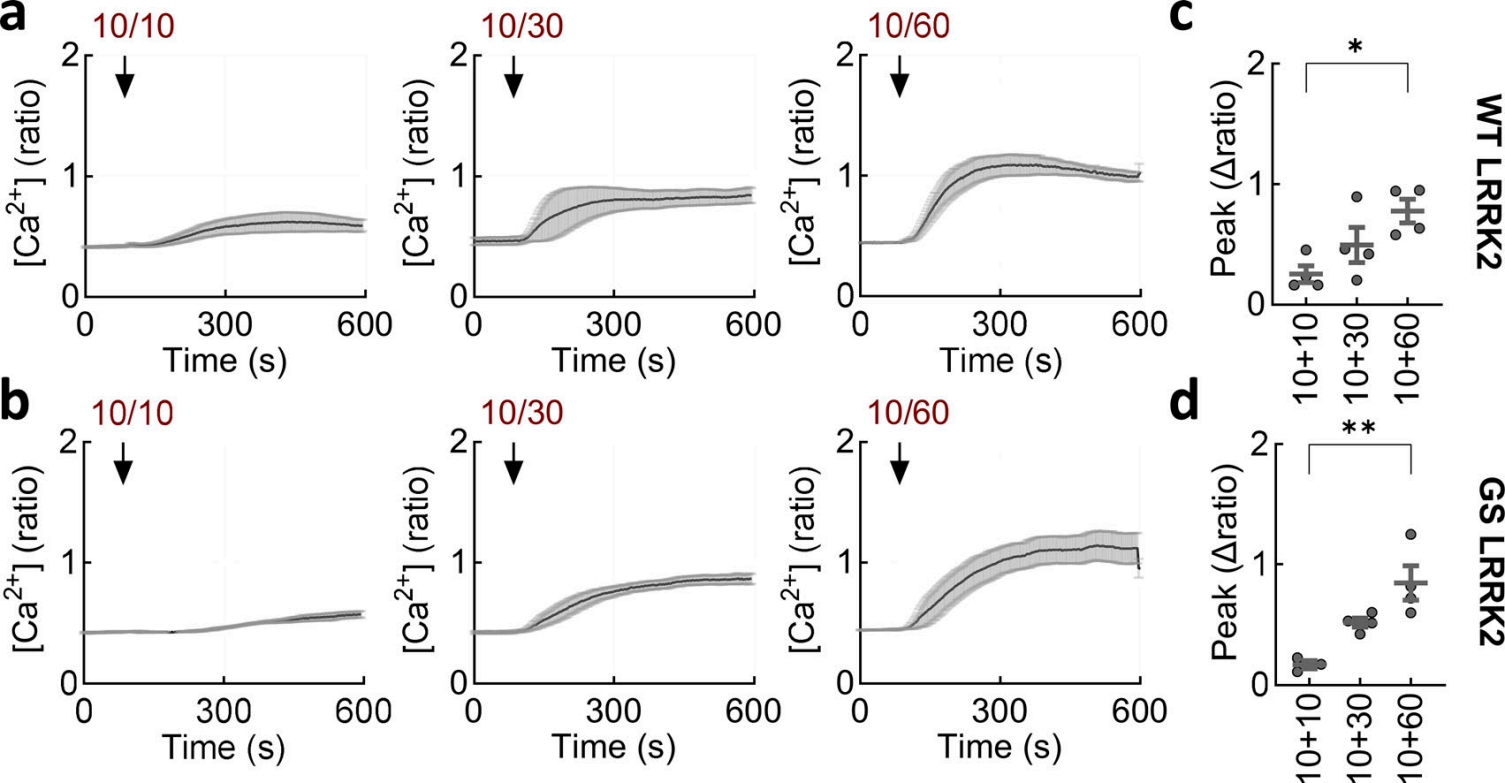
**Effects of TPC2 agonists on cytosolic Ca** ^**2+**^ **in SH-SY5Y cells. (a and b)** Ca^2+^ signals (mean ± SEM, *n* = 3–4 independent biological replicates) in cells expressing wild-type (a) and G2019S (b) LRRK2 in response to TPC2-A1-N (10 µM) in combination with increasing concentrations of TPC2-A1-P (10–60 µM). **(c and d)** Summary data (mean ± SEM from three to four independent biological replicates) quantifying the peak change in the Ca^2+^ signals in cells expressing wild-type (c) and G2019S (d) LRRK2. Each point represents the mean response from a cell population. **P < 0.01, **P < 0.01 (one-way ANOVA, Tukey’s test).

We also examined TPC2 activation in the presence of external Ca^2+^ (Fig. 3 d). Strikingly, the agonist combination evoked a robust Ca^2+^ signal comparable in amplitude to that evoked by depolarization. This effect was specific as external Ca^2+^ only modestly increased the effects of TPC2-A1-P (Fig. 3, c and d). Activation of TPC2 evoked similarly robust Ca^2+^ responses in cells expressing wild-type and G2019S LRRK2 (Fig. 3 f). These effects were concentration-dependent (Fig. S3). To further test specificity, we used the inactive TPC2-A1-N analog, SGA-10 (Fig. 3 a). SGA-10 in combination with TPC2-A1-P had only modest effects on cytosolic Ca^2+^ (Fig. 3, e and f).

Taken together, these data reveal that TPC2-evoked Ca^2+^ release from lysosomes is coupled with Ca^2+^ entry, consistent with a requirement for TPC2 in deviant depolarization-induced Ca^2+^ entry mediated by LRRK2 G2019S.

### Reducing Ca^2+^ permeability of TPC2 corrects deviant LRRK2-mediated Ca^2+^ entry

**Figure 4.**
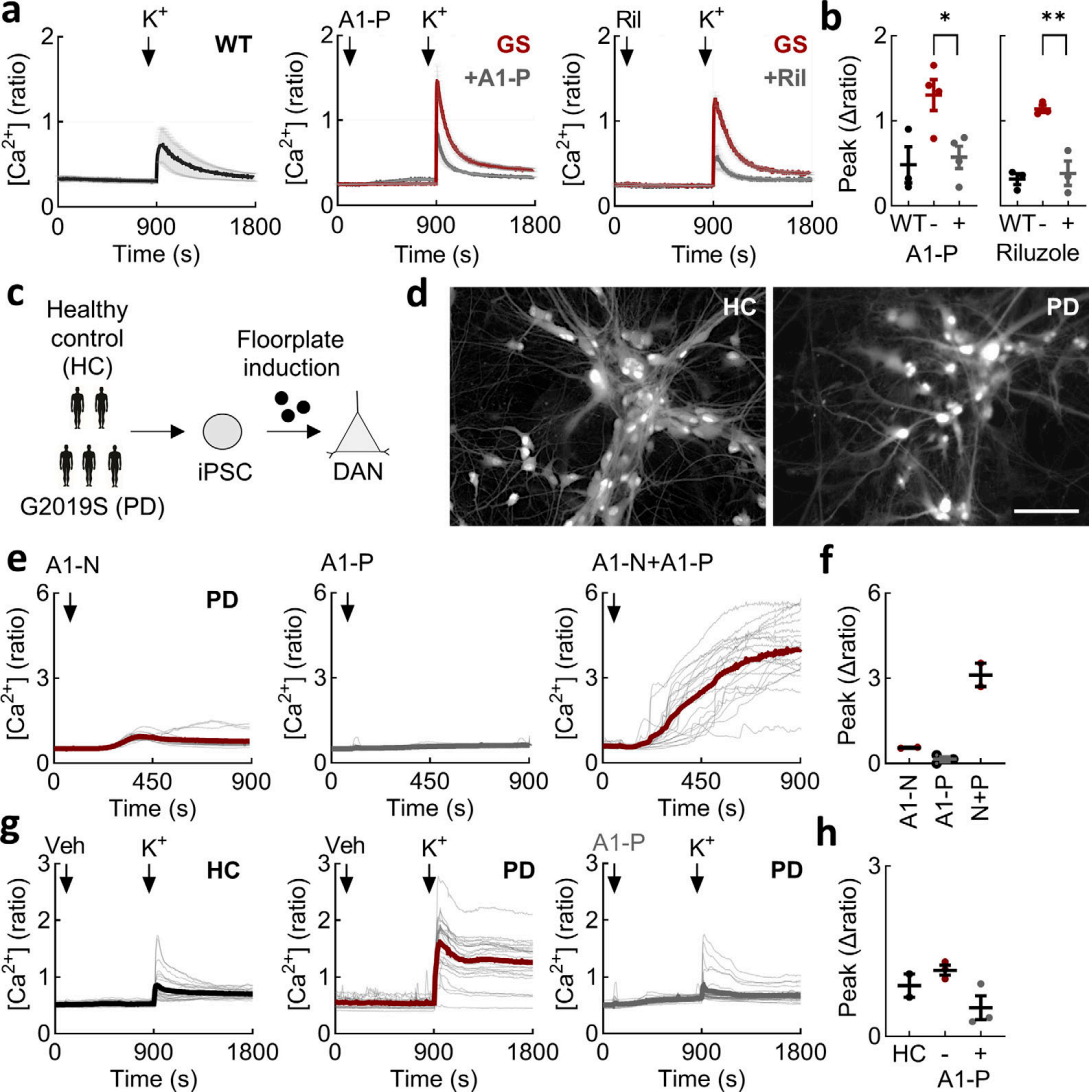
**Reducing Ca** ^**2+**^ **permeability of TPC2 corrects deviant LRRK2-mediated Ca** ^**2+**^ **entry. (a)** Effect of TPC2-A1-P (30 µM) and riluzole (50 µM) on depolarization-evoked Ca^2+^ signals in the indicated cell type (mean ± SEM, *n* = 3–4 independent biological replicates). **(b)** Summary data (mean ± SEM, *n* = 3–4 independent biological replicates) quantifying the peak change in the Ca^2+^ signals from the indicated line and treatment. Each point represents the mean response from a cell population. *P < 0.05, **P < 0.01 (one-way ANOVA, Tukey’s test). **(c)** Schematic of protocol used to generate iPSC-derived midbrain DANs from 3 PD patients carrying the G2019S mutation and two healthy controls (HC). **(d)** Epifluorescence images of live DANs loaded with Fura-2. Scale bar: 100 µm. **(e)** Exemplar Ca^2+^ signals recorded from PD DANs loaded with Fura-2. Cells were stimulated with TPC2-A1-N (30 µM), TPC2-A1-P (30 µM), and a combination of the two. Gray lines are responses from individual cells. The thick lines are the population average. **(f)** Summary data quantifying the peak change in the Ca^2+^ signals from the indicated stimulation. Each point represents the mean response from two to three independent PD lines. **(g)** Exemplar Ca^2+^ signals recorded from healthy and PD DANs loaded with Fura-2. Cells were stimulated K^+^ (50 mM). PD DANs were treated with TPC2-A1-P (30 µM) or vehicle (DMSO) prior to stimulation. Gray lines are responses from individual cells. The thick lines are the population average. **(h)** Summary data (mean ± SEM) quantifying the peak change in the Ca^2+^ signals from the indicated line and stimulation. Each point represents the mean response from 2 independent healthy control lines and three independent PD lines. DANs, dopaminergic neurons.

Activation of TPC2 with TPC2-A1-N increases lysosomal pH and decreases lysosomal motility, whereas channel activation with TPC2-A1-P increases lysosomal exocytosis (Gerndt et al., 2020). Lysosomal exocytosis may represent a route to clear defective lysosomes and their contents. We therefore examined the effect of TPC2-A1-P on depolarization-induced Ca^2+^ signals. TPC2-A1-P was without effect on cytosolic Ca^2+^ levels in LRRK2 G2019S–expressing cells (Fig. 4 a). But, strikingly, TPC2-A1-P reversed the deviant effects of LRRK2 G2019S on depolarization-evoked Ca^2+^ entry (Fig. 4 a). We also tested riluzole, a structurally distinct TPC2 activator with a biophysical current profile similar to TPC2-A1-P (Fig. 3 a) (Zhang et al., 2019). Like TPC2-A1-P, riluzole also reset the Ca^2+^ signals (Fig. 4 a). These data, summarized in Fig. 4 b, show that select activation of TPC2, as well as inhibition (Fig. 3), is corrective.

**Figure S4.**
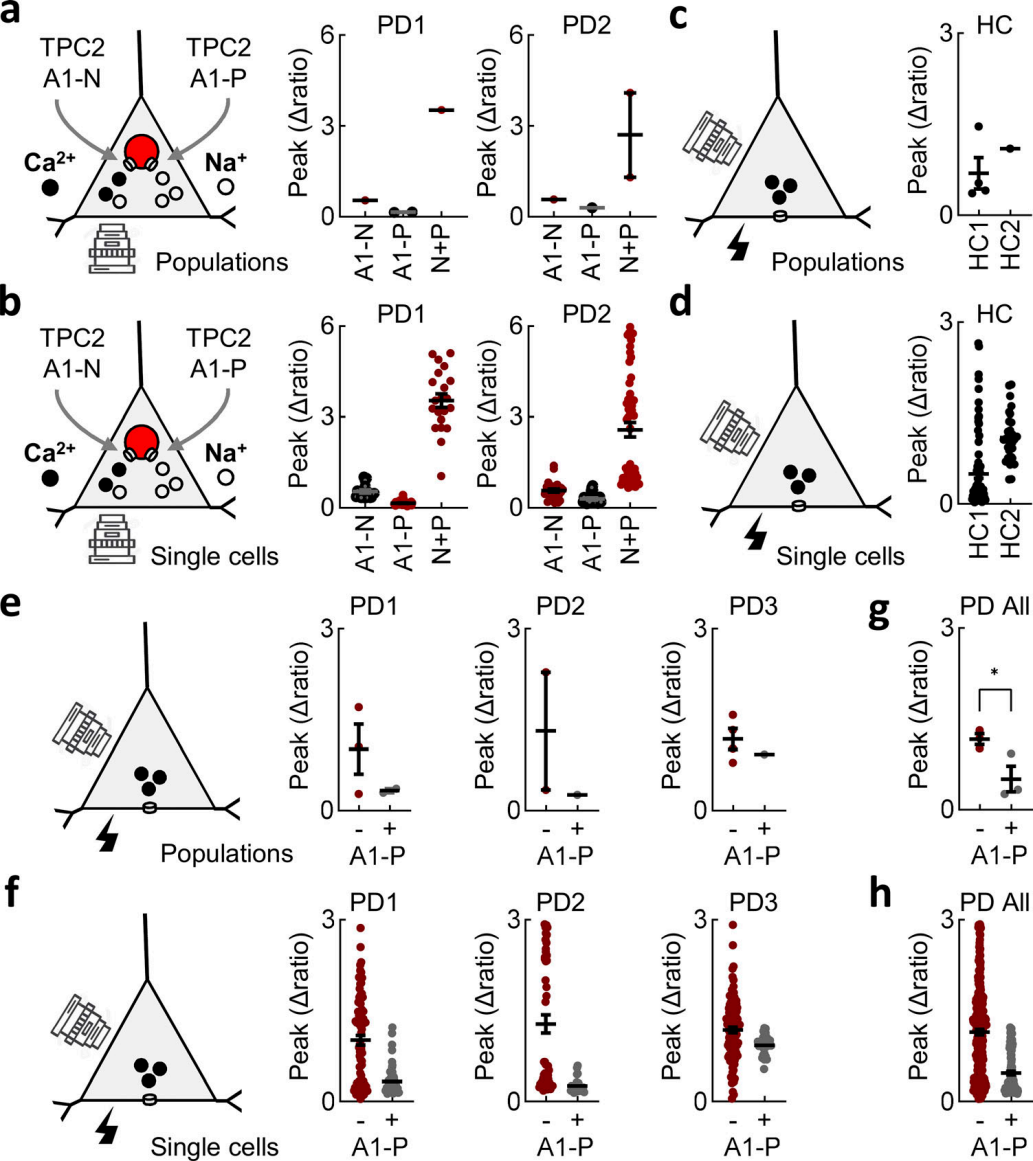
**Effects of TPC2 agonists on cytosolic Ca** ^**2+**^ **in human dopaminergic neurons. (a and b)** Summary data (mean ± SEM from three to four independent biological replicates) quantifying the peak change in the Ca^2+^ signals in two PD lines in response to TPC2-A1-N (30 µM), TPC2-A1-P (30 µM), or a combination of the two. Each point represents the mean response from an independent cell population (a) or single cell (b) separated by line. **(c and d)** Summary data quantifying the peak change in the Ca^2+^ signals in two healthy control lines in response to 50 mM K^+^. Each point represents the mean response from an independent cell population (c) or a single cell (d) separated by line. **(e and f)** Summary data quantifying the peak change in the Ca^2+^ signals in 3 PD lines in response to 50 mM K^+^ treated with TPC2-A1-P (30 µM) or vehicle (DMSO) prior to stimulation. Each point represents the mean response from an independent cell population or a single cell separated by line (e and f) and upon combination (g and h). *P < 0.05 (unpaired *t* test).

Thus far, all experiments were performed in neuroblastoma cells overexpressing LRRK2 (albeit modestly). This model might not recapitulate the situation in diseased neurons. To address this, we generated human midbrain dopaminergic neurons differentiated from PD patient–derived induced pluripotent stem cells (iPSCs; Fig. 4, c and d). These lines are heterozygous for the G2019S mutation. As shown in Fig. 4, e and f, TPC2 agonists acted synergistically to evoke robust Ca^2+^ signals in these neurons just as they did in SH-SY5Y cells. Similar results were obtained in two independent PD lines (Fig. 4 f; and Fig. S4, a and b). Depolarization induced Ca^2+^ signals in both healthy controls and PD lines (Fig. 4 g) albeit variably (Fig. S4, c–f). Importantly, Ca^2+^ signals in PD lines were reduced by TPC2-A1-P (Fig. 4, g and h) just as they were in SH-SY5Y cells, in all three lines tested (Fig. S4, g and h). This analysis extends our findings to human neurons expressing LRRK2 at endogenous levels and validates findings in neuroblastoma cells.

In sum, deviant Ca^2+^ entry evoked by pathogenic LRRK2 can be rescued by biasing cation flux through TPC2 in two independent in vitro models.

### Human TPC2 expressed in *Drosophila* is functional

**Figure 5.**
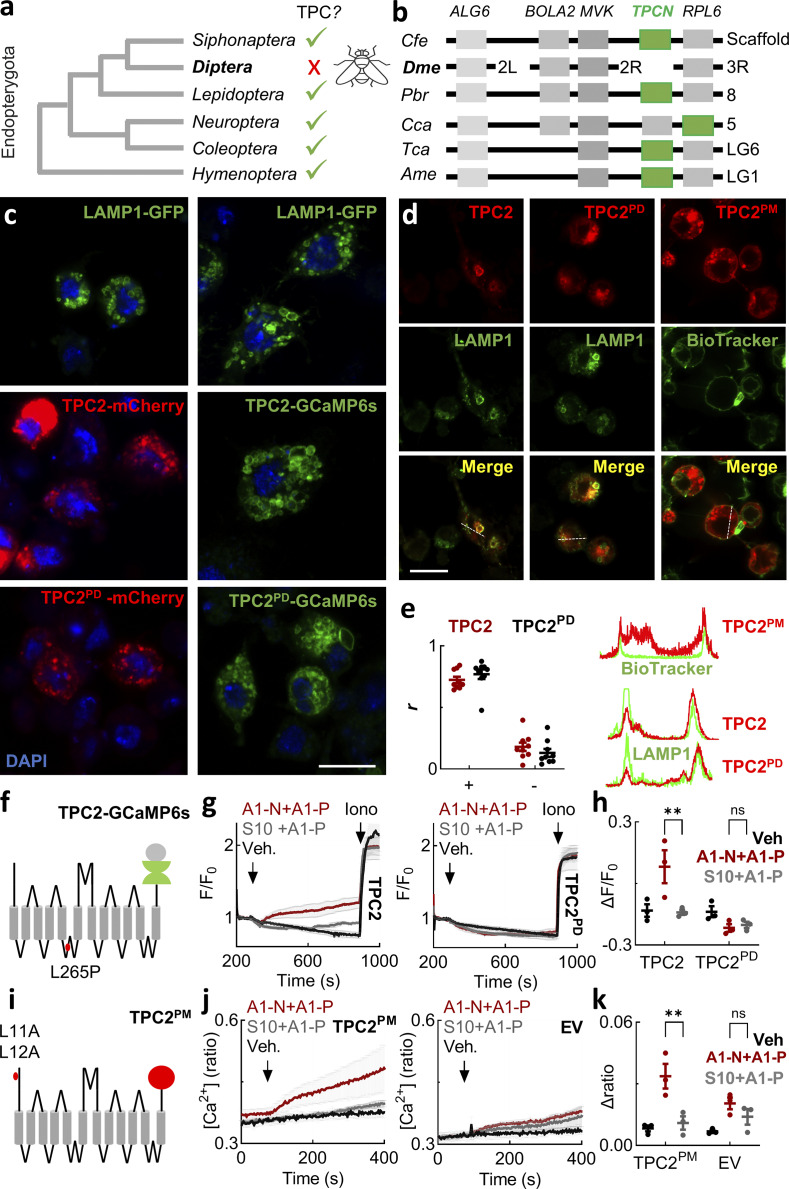
**Human TPC2 expressed in *Drosophila* is functional. (a)** Schematic depicting the absence and presence of TPC homologs in major insect order genomes within Endopterygota. **(b)** Synteny analyses in the vicinity of the *TPCN* gene in the indicated insect. Abbreviations: Cfe, *Ctenocephalides felis*; Dme, *Drosophila melanogaster*; Pbr, *Pieris brassicae*; Cca, *Chrysoperla carnea*; Tca, *Tribolium castaneum*; Ame, *Apis mellifera*. Chromosome assignments are shown to right except for CfeTPC (scaffold, ASM342690v1 CF-g0.4_SCAFFOLD_01886, which is currently unassigned). **(c and d)** Confocal micrographs of *Drosophila* S2R^+^ cells expressing LAMP1-GFP, wild-type or pore-dead TPC2-mCherry and TPC2-GCaMP6s, and TPC2^PM^-mCherry. In c, cells were stained with DAPI. In d, cells coexpressed LAMP1 or were labeled with BioTracker to mark the plasma membrane. Scale bar: 10 µm. **(e)** Colocalization analyses of the indicated TPC2 construct with LAMP1 and BioTracker using Pearson’s correlation coefficient (left) and intensity analysis (right) from the images in d. Negative controls for correlations (−) were obtained upon image rotation. **(f)** Schematic of TPC2 fused at its C terminus with the genetically encoded Ca^2+^ indicator GCaMP6s. **(g)** Ca^2+^ signals (mean ± SEM, *n* = 3 independent biological replicates) in response to TPC2-A1-N (30 µM) or SGA-10 (30 µM) in combination with TPC2-A1-P (30 µM) in cells expressing wild-type (left) or pore-dead TPC2-GCaMP6s (right). Cells were stimulated in the absence of external Ca^2+^ and with ionomycin (10 µM) toward the end of the recording using DMSO as a vehicle (Veh.) control. **(h)** Summary data (mean ± SEM, *n* = 3 independent biological replicates) quantifying the peak change in the Ca^2+^ signals from the indicated cells and treatment. Each point represents the mean response from a cell population. **P < 0.01 (two-way ANOVA, Tukey’s test). **(i)** Schematic of TPC2-mCherry depicting mutations within the N-terminal endo-lysosomal targeting motif to redirect it to the PM. **(j)** Ca^2+^ signals (mean ± SEM, *n* = 3 independent biological replicates) in response to TPC2-A1-N (30 µM) or SGA-10 (30 µM) in combination with TPC2-A1-P (30 µM) in cells expressing TPC2^PM^ (left) or empty vector (right). **(k)** Summary data (mean ± SEM, *n* = 3 independent biological replicates) from quantifying the peak change in the Ca^2+^ signals from the indicated cells and treatment. Each point represents the mean response from a cell population. *P < 0.05, **P < 0.01, ***P < 0.001 (two-way ANOVA, Tukey’s test). PM, plasma membrane.

We next tested whether TPC2 in itself was sufficient to disrupt dopaminergic neuron function. For these experiments, we leveraged the genetic tractability of *Drosophila* in which the G2019S variant of human LRRK2 or its fly equivalent perturbs visual and motor function (Afsari et al., 2014; Cording et al., 2017; Fellgett et al., 2021). As shown in Fig. 5 a, orthologs of the TPC gene (*TPCN*) were not readily identifiable in *Drosophila* and other *Dipterans*. But they were present in a number of insect orders including the closely related *Lepidoptera* and *Siphonaptera*. Synteny analyses showed that the TPC gene when present was in general flanked by *MVK* and *RPL6* (Fig. 5 b). These genes are located on different chromosomes in *Diptera*, indicating that loss of the TPC gene was likely associated with chromosomal rearrangements. Consistent with this, *ALG* and *BOLA1* that are normally upstream neighbors are also located on different chromosomes in *Diptera*. Thus, TPCs have undergone lineage-specific localized loss. This renders *Drosophila* an ideal null background for assessing human TPC2 functionality.

We first expressed human TPC2 tagged with mCherry in *Drosophila* S2R^+^ cells and examined its localization by confocal microscopy. As shown in Fig. 5 c, TPC2 localized to vesicular structures similar to the lysosomal marker LAMP1. The coexpression of TPC2 and LAMP1 confirmed colocalization of the two (Fig. 5, d and e). Similar results were obtained with the pore-dead mutant, TPC2^PD^ (Fig. 5, d and e).

We also expressed TPCs tagged with the genetically encoded Ca^2+^ indicator GCaMP6s (Gerndt et al., 2020) to monitor channel activity (Fig. 5 f). As with the mCherry-tagged constructs, TPC2- and TPC2^PD^-GCaMP6s localized to vesicular structures (Fig. 5 c). Stimulation of TPC2-GCaMP6s with a combination of TPC2-A1-N and TPC2-A1-P resulted in a Ca^2+^ signal, albeit modest relative to ionomycin (Fig. 5 g). This signal was specific because the TPC2-A1-N analog SGA-10 combined with TPC2-A1-P had little effect on GCaMP6s fluorescence (Fig. 5 g). Additionally, as shown in Fig. 5 g, the TPC2 agonist combination did not evoke a response in cells expressing TPC2^PD^-GCaMP6s, but ionomycin responses were similar. These data are summarized in Fig. 5 h.

To further test handling of human TPC2 by *Drosophila* cells, we disrupted the endo-lysosomal targeting motif (Leu11/Leu12) in TPC2 (Fig. 5 i). The expression of TPC2^PM^ (for plasma membrane) tagged with mCherry in S2R+ cells resulted in peripheral and intracellular labeling (Fig. 5 d). Colocalization analyses with a plasma membrane marker confirmed rerouting away from the lysosomes (Fig. 5 e). Ca^2+^ imaging experiments using cells labeled with Fura-2 revealed that rerouted TPC2 responded to the TPC2-A1-N and TPC2-A1-P combination. Again, this signal was specific because SGA-10 when combined with TPC2-A1-N was without effect. And there was little influx in cells expressing the empty vector (Fig. 5, j and k).

Taken together, we show that heterologously expressed human TPC2 traffics and functions normally in *Drosophila* cells, which lack TPC orthologs.

### TPC2 disrupts dopaminergic function in vivo

**Figure 6.**
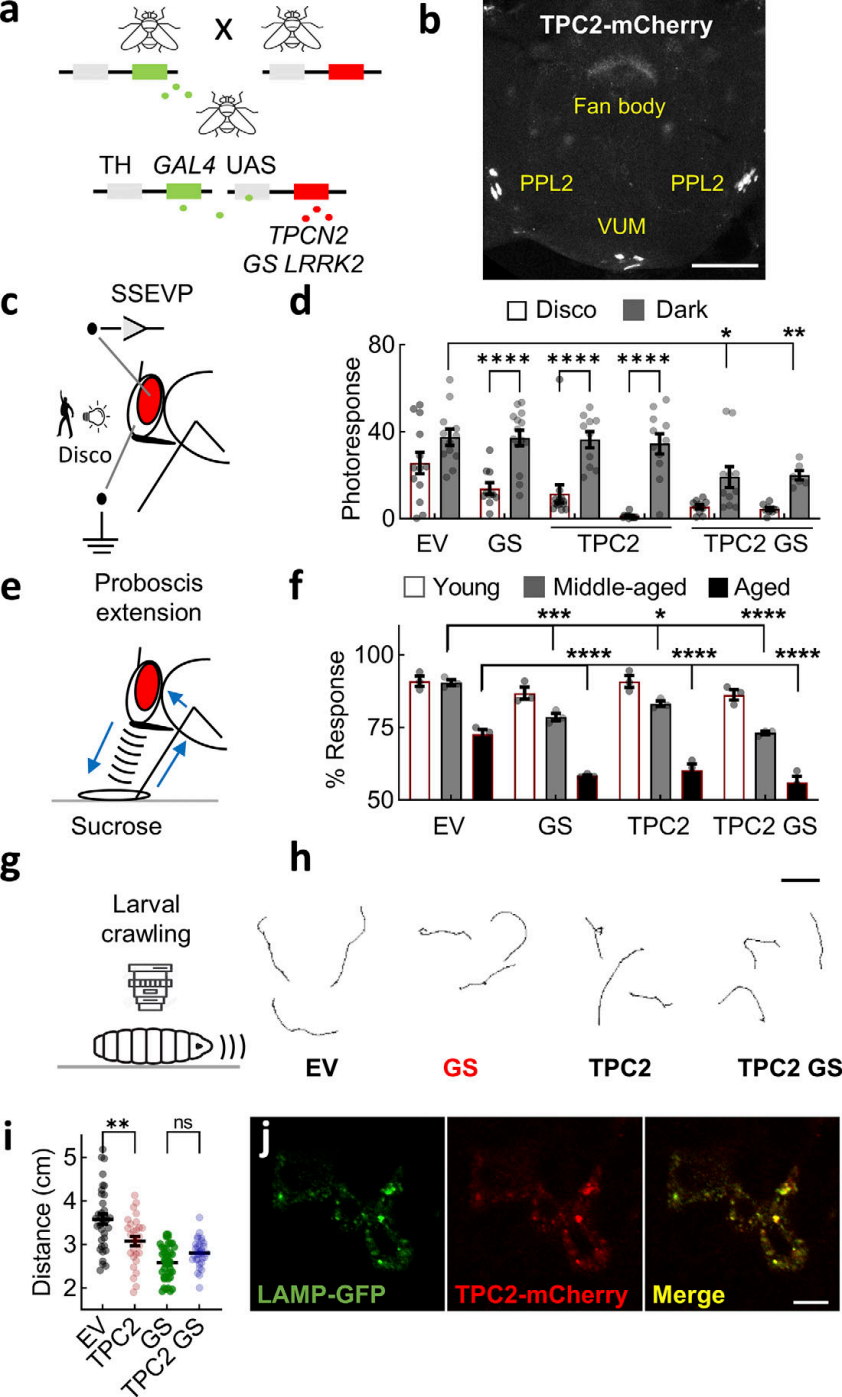
**TPC2 disrupts dopaminergic function in vivo. (a)** Schematic depicting the UAS/GAL4 system to drive the expression of *TPCN2* and/or *LRRK2* G2019S under the control of the TH promoter in *Drosophila*. **(b)** Confocal micrograph of an adult brain showing the expression of TPC2-mCherry in dopaminergic neurons that drive vision (PPL) and movement (VUM). **(c)** Schematic depicting in vivo vision assay to assess dopaminergic function. SSVEP analyses in the retina following mild visual stress induced by repetitive light stimulation resembling a disco. **(d)** Visual responses in lamina neurons (mean ± SEM) from transgenic flies expressing empty vector (*n* = 13), LRRK2 G2019S (*n* = 10), TPC2-mRFP (*n* = 14/11), or both (*n* = 10/10) following disco treatment (mean ± SEM). Flies reared in the dark were used as controls (*n* = 12, 14, 10/11, 11/6 for EV, GS, TPC2, and TPC2 GS, respectively). Each point represents the response from an individual animal. *P < 0.05, **P < 0.01, ***P < 0.001 (two-way ANOVA, Sidak’s test). **(e)** Schematic depicting the PER in response to a sucrose solution to assess dopaminergic-mediated movement in vivo. **(f)** PER in transgenic flies expressing TPC2-mCherry, LRRK2 G2019S, or both (mean ± SEM). Flies were analyzed according to age after enclosure where 1 day was designated “young,” 4 days designated “middle aged,” and 7 days designated “aged.” Each point represents the proportion of responsive animals from an independent trial (*n* = 3). **P < 0.01, ***P < 0.001 (two-way ANOVA, Tukey’s test). **(g)** Schematic depicting crawling of larvae to assess dopaminergic-mediated movement in vivo. **(h)** Exemplar crawling tracks recorded over a 60-s period from lines expressing TPC2-GCaMP6s and/or LRRK2 G2019S. Scale bar: 1 mm. **(i)** Summary data (mean ± SEM) quantifying movement of the indicated single- and double-transgenic larvae (*n* = 20–42). Each point represents the distance traveled by an individual animal. ***P < 0.001, ****P < 0.0001 (one-way ANOVA, Tukey’s test). **(j)** Confocal micrographs of larval brain showing the expression of TPC2-mCherry and LAMP1-GFP in dopaminergic neurons. Scale bar: 10 µm. PER, proboscis extension reflex; TH, tyrosine hydroxylase; EV, empty vector.

Having established the functionality of human TPC2 in *Drosophila* in vitro, we generated transgenic flies expressing human TPC2 in dopaminergic neurons under the tyrosine hydroxylase promoter. We used the bipartite GAL4-UAS system to insert transgenes in the AttP2 genomic landing site (Fig. 6 a) (Bischof et al., 2007). These animals were analyzed in parallel with those expressing LRRK2 G2019S (Fig. 6 a). As shown in the confocal images in Fig. 6 b, TPC2 was readily detectable in tyrosine hydroxylase–positive neurons. This included staining of PPL2 neurons, which control vision, and TH-VUM, which controls movement. To examine the functional consequences of TPC2 expression in intact flies, we analyzed visual responses in the retina and locomotion (Fig. 6 c).

Vision defects are a common nonmotor symptom of PD (Schapira et al., 2017). Visual responses are controlled by dopamine neurons, which ramify the medulla and optic lobe. We used steady-state visual evoked potential (SSVEP) analysis, which separates out the responses of photoreceptors, lamina neurons, and medulla neurons, and highlights any changes in synaptic transmission in the retina (Afsari et al., 2014). Flies were raised in the dark or in a “disco-chamber” where the light is turned on and off at random to induce a mild visual stress (Fig. 6 d). This stress accelerates neurodegeneration (Hindle et al., 2013), thereby mimicking neuronal loss in PD. Flies expressing LRRK2 G2019S exhibited significantly smaller visual responses in the lamina neurons and photoreceptors after disco (Fig. 6 d). In contrast, visual responses were not perturbed in flies raised in the dark (Fig. 6 d). Visual responses in two independent TPC2-expressing lines were also substantially reduced in the light but not in the dark, thereby phenocopying LRRK2 G2019S (Fig. 6 d). We additionally created and analyzed double transgenics expressing both TPC2 and LRRK2 G2019S. Importantly, visual disturbances were readily detected in the double-transgenic flies reared in the dark (Fig. 6 d). Thus, whereas TPC2 and LRRK2 G2019S can mediate dopaminergic dysfunction independently of each other under stressed conditions, they synergize in a more physiologically unstressed setting. TPC2 thus recapitulates the deleterious actions of pathogenic LRRK2 on dopaminergic function in vivo.

**Figure S5.**
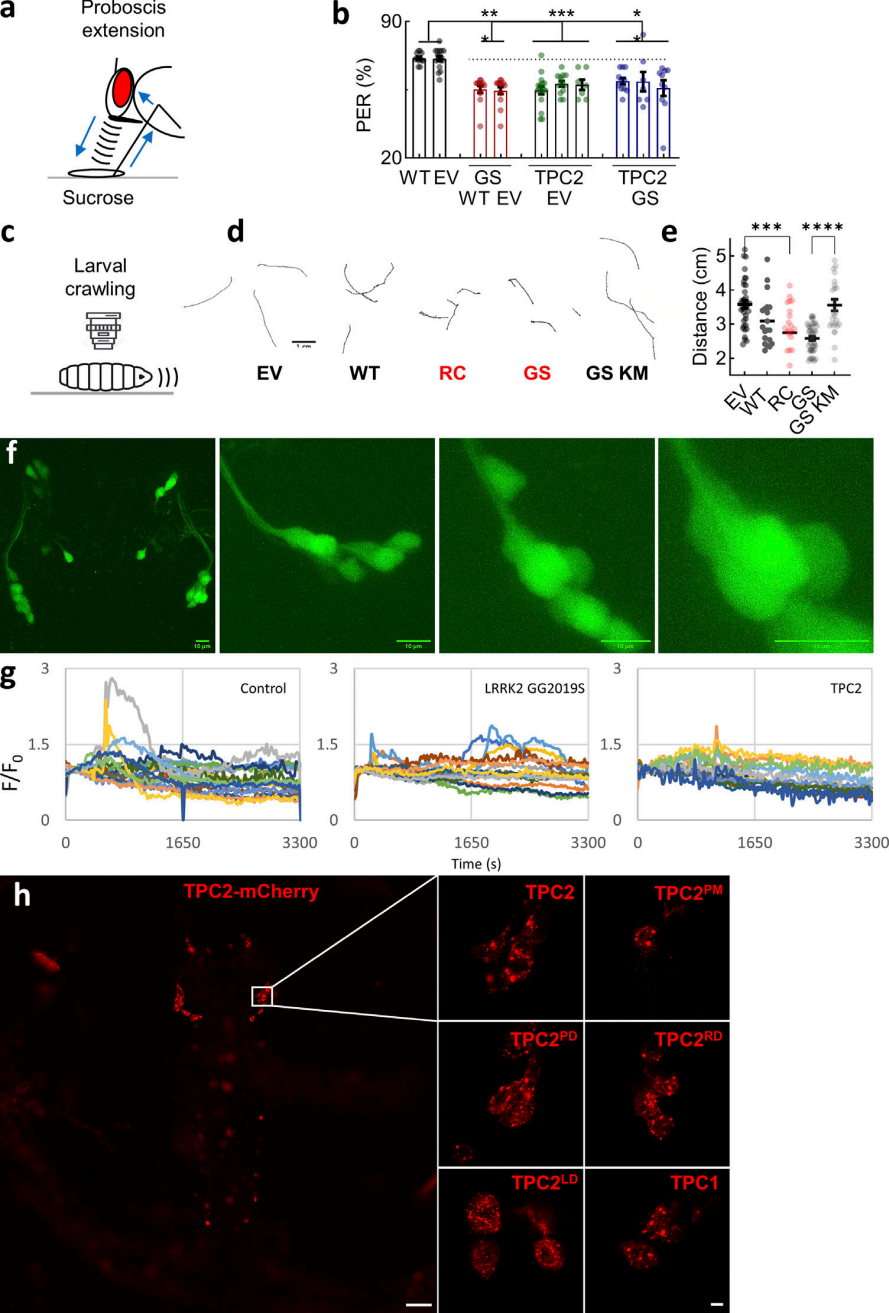
**Effects of TPC2 and LRRK2 expression in *Drosophila* dopaminergic neurons. (a)** Schematic depicting the PER in response to a sucrose solution to assess dopaminergic-mediated movement in vivo. **(b)** Summary data (mean ± SEM) quantifying PER in transgenic flies (7 days) expressing TPC2-mCherry, LRRK2 G2019S, or both (mean ± SEM) in dopaminergic neurons. Each point represents the proportion of responsive animals from an independent line and trial (*n* = 7–17). **P < 0.01, ***P < 0.001 (two-way ANOVA, Tukey’s test). **(c)** Schematic depicting crawling of larvae to assess dopaminergic-mediated movement in vivo. **(d)** Exemplar crawling tracks recorded over a 60-s period from lines expressing the EV, wild-type LRRK2 (WT), R1441C LRRK2 (RC), G2019S LRRK2 without (GS) and with the K1906M kinase–inactivating mutation (GS KM). Scale bar: 1 mm. **(e)** Summary data (mean ± SEM) quantifying movement of the indicated single- and double-transgenic larvae (*n* = 20–42). Each point represents the distance traveled by an individual animal. ***P < 0.001, ****P < 0.0001 (one-way ANOVA, Tukey’s test). **(f)** Two-photon micrographs of an exemplar third instar larval (L3) *Drosophila* brain showing the expression of jGCaMP8m in dopaminergic neurons. Images were acquired at increasing zoom levels (I–IV). Scale bar = 10 µm. **(g)** Spontaneous Ca^2+^ signals recorded from dopaminergic neurons expressing jGCaMP8m from the indicated line. Each trace is a response from an individual neuron. Results are from three independent brain explants for each line. **(h)** Confocal micrographs of transgenic flies expressing mCherry-tagged TPC constructs. Low magnification image (h) showing the expression of TPC2 in the larval brain. Scale bar: 50 µm. Higher magnification images showing the expression of TPC2, the indicated mutant, and TPC1 in DL2 neurons (i). Scale bar: 5 µm. WT, wild type; PER, proboscis extension reflex; EV, empty vector.

Movement was assayed in two ways. In the first, we leveraged the proboscis extension reflex to sucrose, a simple circuit driven by motoneurons that results in the contraction of the proboscis muscle (Fig. 6 e). The circuit is modulated by a single dopaminergic neuron (TH-VUM) that is spontaneously active (much like dopaminergic neurons in the mammalian substantia nigra) and that synapses with the sensory neurons and interneurons (Afsari et al., 2014; Cording et al., 2017; Fellgett et al., 2021). The reflex was assessed by quantifying the proportion of starved flies that extended their proboscis in response to a sugar stimulus. As summarized in Fig. 6 f, nearly all control flies, either young (1 day) or middle aged (4 days), responded to sucrose. This proportion was reduced in aged flies (7 days) to approximately two-thirds. In flies expressing LRRK2 G2019S, locomotor activity was reduced in both middle-aged and aged flies relative to control flies (Fig. 6 f). Essentially, similar results were obtained in transgenic flies expressing TPC2 alone or in combination with G2019S LRRK2 (Fig. 6 f). The proportion of responsive flies was reduced in two independent G2019S-expressing lines and three independent TPC2-expressing lines (Fig. S5, a and b). This defect was particularly prominent in middle-aged double transgenics (Fig. 6 f) pointing to an interaction between LRRK2 and TPC2 as evidenced in our visual assays. Thus, TPC2 again phenocopies and likely synergizes with LRRK2 G2019S to perturb movement in vivo.

The targeted expression of tetanus toxin (Suster et al., 2003) or pathogenic alpha-synuclein (Varga et al., 2014) in dopaminergic neurons inhibits locomotor behavior of larvae. Therefore, in an independent approach to assess movement, we examined the effect of TPC2 and/or LRRK2 G2019S on larval crawling (Fig. 6 g). As shown in the representative tracks in Fig. 6 h, larvae could be readily monitored and locomotion quantified by measuring the distance traveled over 60 s. In larvae expressing LRRK2 G2019S, crawling was reduced relative to control larvae (Fig. 6 h). Similar results were obtained with the R1441C mutation in LRRK2 (Fig. S5, c–e). These effects were specific because the expression of wild-type LRRK2 or kinase-inactive G2019S LRRK2 did not affect crawling (Fig. S5, c–e).

Crawling was also reduced in larvae expressing TPC2 (Fig. 6 h). As with the vision responses to disco, the deleterious effects of LRRK2 G2019S and TPC2 were not additive based on movement in the double-transgenic flies. These data quantified in Fig. 6 i provide further in vivo evidence that TPC2 mimics LRRK2 G2019S. The expression of TPC2 was readily detectable in the larvae by confocal microscopy where it colocalized with LAMP1 (Fig. 6 j). We measured cytosolic Ca^2+^ in brain explants using jGCaMP8m (Fig. S5 f) and resolved spontaneous activity in dopaminergic neurons from control larvae and larvae expressing LRRK2 G2019S and TPC2 (Video 1 and Fig. S5 g). These signals are consistent with autonomous activity in the larval brain.

**Video 1.** **Time lapse (60 min) showing spontaneous Ca** ^**2+**^ **signals recorded from dopaminergic neurons in *Drosophila* larval brains expressing jGCaMP8m from the indicated line.**

Taken together, we show that TPC2, like LRRK2 G2019S, is sufficient to disrupt dopaminergic circuits underpinning both vision and movement in vivo.

### TPC2 defects in vivo require Ca^2+^ channel activity in the lysosome and Rab interactivity

**Figure 7.**
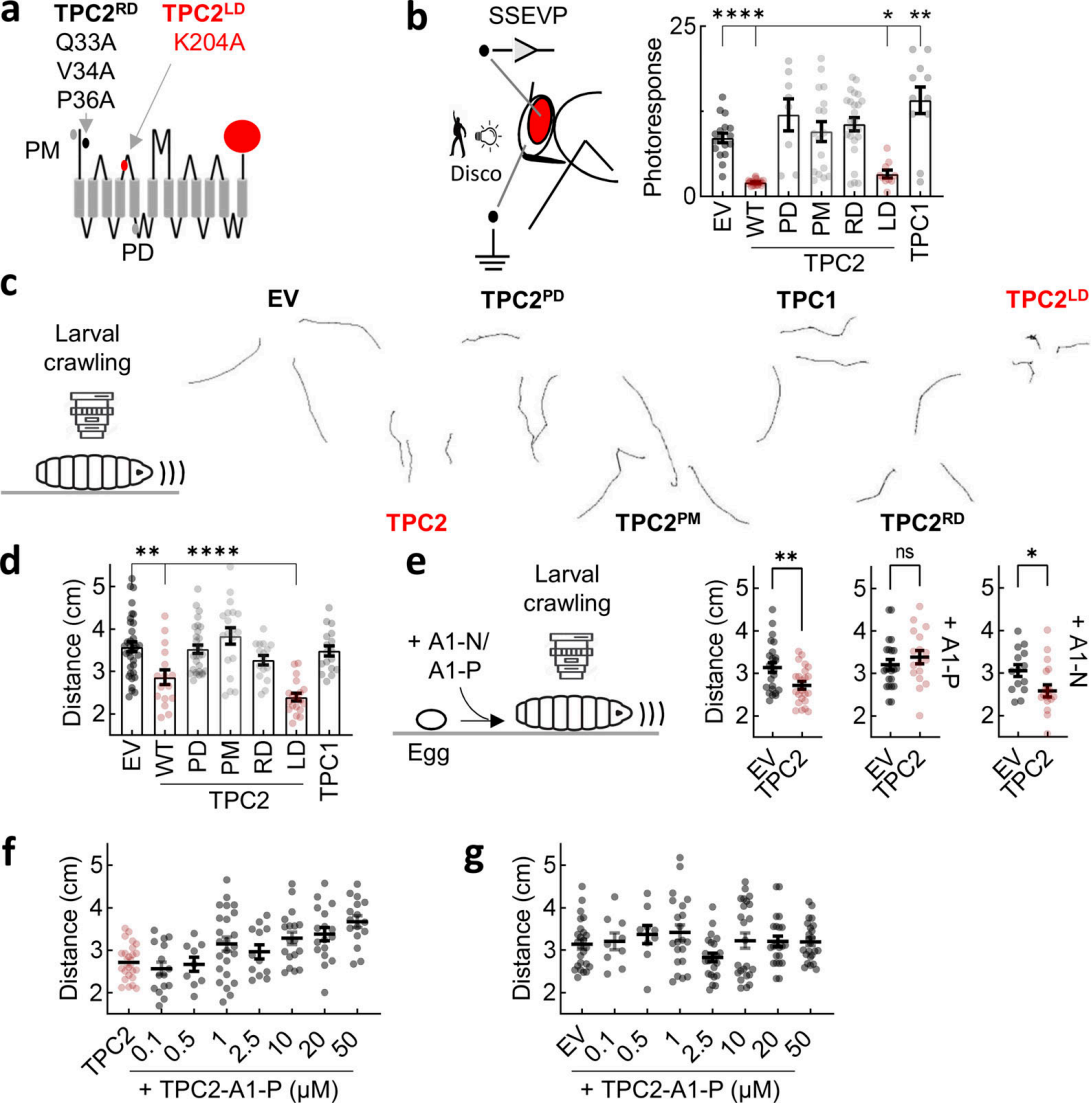
**TPC2 defects in vivo require Ca** ^**2+**^ **channel activity in the lysosome and Rab interactivity. (a)** Schematic of TPC2-mCherry depicting mutations within the N-terminal Rab binding motif to disrupt Rab interaction (RD) and the S4–S5 linker to disrupt lipid binding (LD). Mutations within the pore (PD) and endo-lysosomal targeting motif (PM) are also highlighted. **(b)** Visual responses in lamina neurons from transgenic flies expressing mCherry-tagged WT TPC2, the indicated mutants, and TPC1 following disco treatment (mean ± SEM, *n* = 8–24). Flies expressing the EV were used as controls. Each point represents the response from an individual animal. *P < 0.05, **P < 0.01, ***P < 0.001 (one-way ANOVA, Dunnett’s test). **(c)** Exemplar crawling tracks from transgenic flies expressing mCherry-tagged WT TPC2, the indicated mutants, and TPC1. **(d)** Summary data (mean ± SEM, *n* = 16–36) quantifying movement of the indicated transgenic larva. Larvae expressing the EV were used as controls. Each point represents the distance traveled in 60 s by an individual animal. **P < 0.01, ****P < 0.0001 (one-way ANOVA, Dunnett’s test). **(e–g)** Effect of TPC2 agonists on movement. Transgenic flies expressing EV or TPC2-GCaMP6s were fed with 20 μM TPC2-A1-N or TPC2-A1-P (e) or increasing concentrations of TPC2-A1-P (f and g). Data (mean ± SEM) were acquired 5 days after feeding where each point represents the response from an individual animal. *P < 0.05, **P < 0.01 (unpaired *t* test). WT, wild type; PM, plasma membrane; RD, rab-deaad; LD, lipid-dead; EV, empty vector.

To explore the underlying mechanisms underpinning TPC2-dependent dopaminergic neuron dysfunction in vivo, we generated flies expressing a series of TPC2 mutants (Fig. 7 a) that included the pore-dead and plasma membrane–targeted variants.

The corresponding visual responses are shown in Fig. 7 b. These analyses revealed that whereas wild-type TPC2 perturbed vision, the pore-dead mutant that is demonstrably inactive in vitro (Fig. 5) did not. These data indicate that channel activity is required for TPC2 action in vivo. To mitigate against nonspecific effects of channel activity, we took two approaches. In the first, we leveraged TPC2 rerouted to the plasma membrane, which is demonstrably active but mistargeted (Fig. 5). In the second, we examined the effects of TPC1, which is closely related to TPC2 but expressed in earlier compartments of the endo-lysosomal system (Brailoiu et al., 2009b). Both TPC2^PM^ and TPC1 failed to disrupt vision in vivo (Fig. 7 b). These data suggest that dopaminergic dysfunction results from aberrant channel activity specifically on the lysosome.

Much evidence implicates Rab proteins in LRRK2 action, and TPC2 is a Rab interactor (Lin-Moshier et al., 2014). We therefore analyzed TPC2 in which the Rab binding site within the N terminus of TPC2 was mutated (Fig. 7 a). This mutant (TPC2^RD^, for Rab-dead) failed to perturb dopaminergic function (Fig. 7 b), suggesting that the inhibitory effects of TPC2 require Rab interactivity. Activation of Na^+^ currents through TPC2 by PI(3,5)P_2_ proceeds through residues in the S4-S5 linker. We therefore also analyzed the effect of mutating Lys 204 (Fig. 7 a), which is PI(3,5)P_2_-insensitive (She et al., 2019). TPC2^LD^ (for lipid-dead) disrupted vision similar to wild-type TPC2 (Fig. 7 b), suggesting that the effects of TPC2 are PI(3,5)P_2_-independent and thus likely driven by Ca^2+^.

In an independent approach, we examined the effects of the TPC2 mutants on larval crawling (Fig. 7 c). Similar to the vision assays, the TPC2 pore and targeting mutants were without effect on movement (Fig. 7 d). So too were TPC1 and the TPC2 Rab-dead mutant (Fig. 7 d). In further analogy with the effects on vision, the lipid-dead TPC2 mutant produced inhibitory effects (Fig. 7 c). Thus, there was marked congruence in the neurophysiological and behavioral phenotypes whereby TPC2 but not TPC1 Ca^2+^ channel activity in the lysosome disrupts vision and movement in a Rab-dependent manner. Confocal microscopy of larval brains confirmed the expression of TPC2 in all lines (Fig. S5 h).

Finally, we examined whether chemically targeting TPC2 could improve dopaminergic function in vivo. For these experiments, larvae expressing TPC2 were fed with TPC2-A1-P, which reverses Ca^2+^ defects in vitro (Fig. 4), and its effect on crawling was assessed. As shown in Fig. 7 e, including TPC2-A1-P in food through development reversed the inhibitory effects of TPC2 on movement at the larval stage. In contrast, feeding with TPC2-A1-N did not (Fig. 7 e). The effect of TPC2-A1-P on crawling was dose-dependent (Fig. 7 f). The normal absence of TPC2 in *Drosophila* allowed us to directly probe off-target effects of TPC-A1-P. As shown in Fig. 7 f, TPC2-A1-P had little effect in control larvae firmly attesting to specificity. Thus, dopaminergic dysfunction induced by TPC2 in vivo can be reversed by pharmacologically targeting TPC2 in an agonist-selective manner.

Overall, we provide mechanistic insight into and a chemical reversal strategy for TPC2-dependent dopaminergic dysfunction in vivo.

## Discussion

Here, we identified Ca^2+^, neurophysiological, and behavioral defects mediated by TPC2 and showed that targeting this channel reversed phenotypes mediated by a PD-relevant mutation in LRRK2.

Our data revealed that voltage-gated Ca^2+^ entry is increased upon mutation of LRRK2 in a model dopaminergic neuron (Fig. 1), as well as iPSC-derived midbrain dopaminergic neurons (Fig. 4). Changes in voltage-gated Ca^2+^ channel expression in PD have been known for some time (Hurley and Dexter, 2012). More recent studies have identified increased Ca^2+^ levels and increased voltage-gated Ca^2+^ currents in LRRK2 G2019S–expressing dopaminergic neurons derived from iPSCs and knock-in mice resulting from increased translation (Kim et al., 2020, 2021). Enhanced evoked Ca^2+^ entry in the presence of the LRRK2 G2019S mutation reported here supports these findings. Indeed, growing literature identifying defects in iPSC-derived dopaminergic neurons is consistent with a cell-autonomous role for LRRK2 in regulating neuronal activity (Bono et al., 2021; Carola et al., 2021; Kim et al., 2020). Our data, however, contrast results in iPSC-derived sensory neurons where K^+^-induced Ca^2+^ entry is reduced by the G2019S mutation (Schwab and Ebert, 2015) and in a mixed iPSC-derived neuronal population where K^+^-induced Ca^2+^ entry is enhanced but only after depletion of endoplasmic reticulum Ca^2+^ stores (Korecka et al., 2019). This suggests the LRRK2-induced defects may manifest in a cell type–specific manner. Enhanced Ca^2+^ entry was unlikely to be due to compromised endoplasmic reticulum Ca^2+^ homeostasis or cytoplasmic buffering because neither the Ca^2+^ signals evoked upon endoplasmic reticulum Ca^2+^ depletion nor those upon subsequent Ca^2+^ entry were affected upon LRRK2 mutation. Our data, using a number of chemical and molecular interventions, including the use of two chemically distinct permeabilizing agents (Figs. 2 and S2) (Atakpa et al., 2019; Morgan et al., 2020; Yuan et al., 2021), instead identified a prominent role for lysosomes and TPC2 in mediating Ca^2+^ defects at the plasma membrane.

In hippocampal neurons, voltage-gated Ca^2+^ influx is functionally coupled with NAADP-mediated lysosomal Ca^2+^ release (Padamsey et al., 2017). Thus, as reported here, antagonizing NAADP action reduced postdepolarization-induced Ca^2+^ influx. Interestingly, this interplay was coupled with lysosomal exocytosis to maintain dendritic morphology (Padamsey et al., 2017)—a key process regulated by LRRK2 (MacLeod et al., 2006) and NAADP (Brailoiu et al., 2005). Further evidence that lysosomal Ca^2+^ release and external Ca^2+^ entry are coupled comes from our data using recently described TPC2 agonists to directly activate TPC2 (Gerndt et al., 2020). We show that the NAADP-mimetic TPC2-A1-N evokes Ca^2+^ signals with a major Ca^2+^ entry component when combined with a PI(3,5)P_2_ mimetic (which alone does little to Ca^2+^) (Figs. 3 and 4). Such synergism adds to similar effects of TPC2 activation observed in cancer cells and pancreatic acinar cells serving as a key functional diagnostic of endogenous TPC2 channels (Yuan et al., 2022). Local NAADP-evoked Ca^2+^ signals have been reported to depolarize medulla neurons (Brailoiu et al., 2009b). Thus, lysosome–plasma membrane crosstalk is likely bidirectional with Ca^2+^ entry evoking lysosomal Ca^2+^ release and lysosomal Ca^2+^ release evoking Ca^2+^ entry. More work is required to understand exactly how TPC2 and voltage-gated Ca^2+^ channels communicate within this novel axis. Efforts at targeting Cav1.3 are underway in PD (Caulfield et al., 2023). We suggest that targeting TPC2, perhaps in combination, also be considered.

TPCs are ancient ion channels that can be traced back to unicellular organisms (Li et al., 2021; Rahman et al., 2014). But there has been lineage-specific loss of TPC isoforms in mammals (Brailoiu et al., 2010a; Cai and Patel, 2010) and, as reported here, complete loss in some insects (Fig. 5). The absence of TPCs in *Drosophila* appears to be a result of chromosomal fragmentation and an exception rather than the rule in insects since TPCs were identifiable in other insect orders. We leveraged this loss to study TPC2 heterologously expressed in a “clean” background and importantly its pharmacology in a genetically tractable way. But it should be noted that we cannot rule out functional compensation of TPC loss by other lysosomal channels. Our data show that the expression of TPC2 in dopaminergic neurons disrupted vision and movement, thereby phenocopying pathogenic LRRK2. Our in vivo electrophysiological assays centered around visual stress evoked by repetitive light episodes (“disco”). But the dark controls proved informative too as TPC2 and LRRK2 synergized to disrupt dopaminergic function, thereby placing the two proteins in a common pathway in the eye. Notably, we established crawling assays in flies expressing TPC2 and LRRK2 G2019S (or both) and found locomotion defects (Fig. 6). At present, we know little about the function of TPCs in a neuronal context but our studies add to recent ones implicating them in synaptic plasticity (Foster et al., 2018) and social behavior (Martucci et al., 2023) building on early work identifying a role for NAADP in neurotransmission (Brailoiu et al., 2003) and neuronal differentiation (Brailoiu et al., 2006).

To explore the mechanisms underpinning TPC2-mediated defects, we translated point mutations in TPC2 with defined action at the channel level through to dopaminergic deficits in vivo (Fig. 7). Our data revealed five salient features: first, TPC2 disruptions were pore-dependent consistent with reversal of LRRK2 Ca^2+^ defects by the same mutant (Fig. 2). Second, the effects required localization to the lysosome, indicating the highly localized fluxes as the driver as opposed to nonspecific channel activity. Third, defects were specific for TPC2 over TPC1 further attesting to specificity and further suggesting localized activity given that TPC1 is targeted to more distal compartments of the endo-lysosomal system than TPC2 (Brailoiu et al., 2009a). Fourth, disruption may require Rab interactivity. The Rab family of trafficking proteins are of particular relevance to LRRK2 signaling (Kuwahara and Iwatsubo, 2020). Similar to TPC2, LRRK2 reportedly interacts with a number of these molecular switches including Rab5 (Shin et al., 2008), Rab7 (Dodson et al., 2012), and Rab7L1/Rab29 (MacLeod et al., 2013). The identification of Rab8 and Rab10 as direct LRRK2 substrates (Steger et al., 2016) further implicates LRRK2 in regulating endo-lysosomal morphology and function (Eguchi et al., 2018). Fifth, disruption was independent of PI(3,5P)_2_ binding. PI(3,5)P_2_-mediated Na^+^ signaling is abolished by the K204A mutation, but this did not prevent functional deficits. Thus, Na^+^ flux is probably not relevant here pointing instead to Ca^2+^ as the culprit. Recent work has shown that the mutation of K204 and other residues that form the PI(3,5P)_2_ binding site also block NAADP activation (Saito et al., 2023). NAADP normally induces Ca^2+^ flux through TPC2 via NAADP binding proteins (Gunaratne et al., 2021, 2023; Marchant et al., 2022). Thus, TPC2^LD^ might be considered “ligand-dead” as opposed to lipid-dead, meaning that it is constitutive leak of Ca^2+^, not evoked flux through TPC2, which is (dys)functionally relevant. We found that various mutants had essentially indistinguishable results in our vision and movement assays. We therefore propose this approach as a novel and robust systems-level opportunity for interrogating TPC2 functionality.

Ostensibly, the most interesting finding reported here is reversal of defects by targeting of TPC2. For LRRK2-Ca^2+^ defects, antagonizing NAADP action and blocking the TPC2 pore either chemically or molecularly were sufficient to reset Ca^2+^ signals (Fig. 2). These data extend previous work in fibroblasts from LRRK2 G2019S patients where lysosomal morphology defects were reversed by targeting of TPC2 but not TPC1 (Hockey et al., 2015) to a neuronal setting. But equally, activating TPC2 with TPC2-A1-P also reset deviant Ca^2+^ signals, both in neuroblastoma cells overexpressing LRRK2 G2019S and in iPSC-derived dopaminergic neurons expressing pathogenic LRRK2 at endogenous levels (Fig. 4), as well as movement defects in vivo (Fig. 7). This was despite TPC2-A1-P synergizing with TPC2-A1-N to mediate Ca^2+^ influx (Figs. 3 and 4). TPC2-A1-P was shown recently to reverse lysosomal defects in iPSC models of several lysosomal storage disorders and an in vivo mouse model of Mucolipidosis IV (Scotto Rosato et al., 2022). Given the link between lysosomal storage diseases and PD, we speculate that lysosomal Ca^2+^ is a common defect, or, in the very least, a common target to improve well-being in these disorders. Lysosomal Ca^2+^ defects were reported in GBA1-linked PD (Kilpatrick et al., 2016) building on foundational studies in models of Niemann–Pick type C disease (Lloyd-Evans et al., 2008). Kufor–Rakeb syndrome is a PD-like syndrome caused by mutation of a *ATP13A2* (Ramirez et al., 2006), which encodes a lysosomal ATP-dependent pump with a possible role in Ca^2+^ transport (Narayanaswamy et al., 2019). Targeting TPC2 as described here may thus be of broader benefit in tackling PD. Beyond PD, lysosomal Ca^2+^ defects have been noted in models of Alzheimer’s disease and inhibiting TPC2 provides benefit (Tong et al., 2022). We suggest that selective biasing of TPC2 away from deleterious Ca^2+^ fluxes might have less side-effects than pan-inhibition and propose that such a strategy might find utility in tackling neurodegeneration.

## Materials and methods

### Cells

SH-SY5Y cells were maintained in 1:1 mixture of DMEM and Ham’s F12 medium, supplemented with 10% vol/vol fetal bovine serum (FBS), 100 U/ml penicillin, 100 µg/ml streptomycin, and 1% nonessential amino acids (all from Gibco) at 37°C in a humidified 5% CO_2_ atmosphere. Stable cell lines expressing wild-type LRRK2, pathogenic LRRK2 G2019S, and kinase-inactive LRRK2-K1906A/D1994A/D2017A were generated as described previously (Papkovskaia et al., 2012). Differentiation was induced 24 h after plating by the addition of 10 µM retinoic acid (from Sigma-Aldrich) to 1:1 mixture of DMEM and Ham’s F12 medium supplemented with 2% vol/vol FBS, 100 U/ml penicillin, and 100 µg/ml streptomycin (all from Gibco). Cells were differentiated for 5–7 days.

iPSC lines were obtained from Parkinson’s Progression Markers Initiative. iPSCs were generated from peripheral blood mononuclear cells obtained from healthy controls and people with PD carrying the LRRK2 G2019S mutation (Table S1). Cells were cultured in Essential 8 media (Thermo Fisher) on truncated human vitronectin (Thermo Fisher) in 6-well plates.

S2R^+^ cells (obtained from the *Drosophila* Genomics Resource Centre, Indiana University, Bloomington, IN, USA) were maintained in Schneider’s *Drosophila* medium supplemented with 10% FBS, 100 U/ml penicillin, and 100 µg/ml streptomycin (all from Gibco) at 25°C as semi-adherent cultures.

Cells were plated onto either a 6-well plate, a T25 flask, or 13-mm glass coverslips coated with 20 µg/ml poly-L-lysine (Sigma-Aldrich) in a 24-well plate for qPCR analysis and imaging. For automated Ca^2+^ measurements, cells were plated onto opaque-walled 96-well microplates (Corning).

### Quantitative PCR and western blotting

RNA was extracted using RNeasy Mini Kit and RNase-free DNase Set (both from Qiagen). cDNA was synthesized using Superscript III Reverse Transcriptase (Invitrogen), Random Primers (Promega), and Oligo dT(12-18) primers (Invitrogen). PCR was performed using SYBR Green JumpStart Taq Ready Mix (Sigma-Aldrich) and primers for human LRRK2 and the housekeeping gene, UBC. Samples were denatured for 2 min at 94°C, followed by 40 cycles of denaturation (15 s at 94°C), annealing (30 s at 60°C), and extension (30 s at 72°C). LRRK2 expression was normalized to UBC expression. Details of the primers used are listed in Table S2. Western blotting (Papkovskaia et al., 2012) was performed using antibodies to LRRK2 (1:1,000, ab133518; Abcam) and cathepsin D (1:1,000, ab6313; Abcam) (Papkovskaia et al., 2012).

### Plasmids and transfection

Mammalian expression plasmids encoding GECO1.2 (Zhao et al., 2011), TPC2^L265P^-GFP (Brailoiu et al., 2010b), and LAMP1-GFP (Falcón-Pérez et al., 2005) were described previously.

Insect expression plasmids encoding TPC2 were based on pUASTattB (Bischof et al., 2007). For TPC2-GCaMP, the coding sequence of human TPC2 fused to GCaMP6s (Gerndt et al., 2020) was amplified by PCR using the primers described in Table S3, and the product was inserted into the EcoRI and XbaI sites of pUASTattB. TPC2^L265P^-GCaMP was generated by site-directed mutagenesis of pUASTattB TPC2-GCaMP using the primers described in Table S3. For TPC2-mCherry, the coding of sequence of TPC2 GFP in pCS2+ (Brailoiu et al., 2009a) was directly inserted into the EcoRI and XbaI sites of pUASTattB and the GFP coding sequence at the XhoI and XbaI sites replaced with mCherry. The coding sequences of TPC2^L265P^ (Brailoiu et al., 2010b), TPC2^L11A/L12A^ (Brailoiu et al., 2010b), and TPC2^Q33A/V34A/P36A^ (Lin-Moshier et al., 2014) were directly inserted into the EcoRI and XhoI sites of pUASTattB TPC2-mCherry to form the corresponding mCherry-tagged constructs. TPC2^K204A^-mCherry was generated from TPC2-mCherry by site-directed mutagenesis using the primers described in Saito et al. (2023). For TPC1-mCherry, the coding sequence of TPC1 (Brailoiu et al., 2009a) was amplified by PCR using the primers listed in Table S3, and the product was inserted into the EcoRI and NotI sites of pUASTattB. mCherry was inserted at the NotI and XbaI sites. *Drosophila* LAMP fused to GFP was described in Minin et al. (2006).

SH-SY5Y cells were transfected using Lipofectamine 2000 (Invitrogen) according to the manufacturer’s protocol and used 12–24 h after transfection. S2R^+^ cells were transfected using the Effectene transfection reagent kit (Qiagen) according to the manufacturer’s instructions and used 24–48 h after transfection. GAL4-dependent UAS expression plasmids were cotransfected at a 1:1 ratio with a pWAGal4 (Kuranaga et al., 2006) (a kind gift from Dr. Nic Tapon, Francis Crick Institute, London, UK).

### Generation of human midbrain dopaminergic neurons

iPSCs were differentiated into midbrain dopaminergic neurons using the floor-plate protocol described in Kriks et al. (2011).

Briefly, iPSCs were passaged with Accutase to yield a single-cell suspension and seeded at 1 × 10^6^ cells/ml in STEMdiff SMADi Neural Induction Media (Stemcell Technologies) supplemented with 10 mM Y27632 (Abcam) on Geltrex LDEV-Free Reduced Growth Factor Basement Membrane Matrix (Thermo Fisher). Media were changed daily for 15 days with one passage at day 6 or 7. Midbrain floor-plate neural precursor cells were then generated by passaging neuroepithelial cells with Accutase and resuspending in 1:1 mixture of N2 media (DMEM:F12 media containing 1X N2 supplement, 1X nonessential amino acids, 10 mM β-mercaptoethanol, 5 mg/ml insulin) and B27 media (Neurobasal media containing 1X B27 supplement, 1X GlutaMAX). The N2:B27 medium mix was supplemented with 20 ng/ml BDNF, 100 ng/ml FGF8, 1 ng/ml TGFβ3 (all from PeproTech), 0.1 mM compound E (Abcam), 200 mM ascorbic acid, and 0.5 mM dibutyryl cAMP (Enzo Lifesciences). Cells were seeded at 0.75 × 10^6^ cells/ml on Geltrex-coated plates and media changed daily for 4 days; and then every other day (no FGF8) for a further 4 days. Neural precursor cells were then passaged with Accutase and seeded for terminal differentiation at 0.125 × 10^6^ cells/ml on polyornithine and laminin (1 mg/ml)-coated coverslips in BrainPhys neuronal media containing SM1 (Stemcell Technologies), and 1X N2 supplement, and supplemented fresh with 20 ng/ml BDNF, 20 ng/ml GDNF, 1 ng/ml TGFβ3, 0.1 mM compound E, 40 mM ascorbic acid, and 0.5 mM dibutyryl cAMP. Cells were cultured for 8 wk with half-medium changes every 2 or 3 days.

### Database mining and genomic synteny

The genomic locations of insect TPC genes were identified using TBlastN searches (Altschul et al., 1997) with the TPC protein sequence from *Ctenocephalides felis* (XP_026472147). The initial screening of syntenic chromosomal regions surrounding TPC homologs was made among the genomes of *Ctenocephalides felis*, *Bombyx mandarina*, *Ceratina calcarata*, and *Pieris brassicae*. The genes of 60S ribosomal protein L6 (*RPL6*), mevalonate kinase (*MVK*), bolA-like protein 2 (*BOLA2*), and dolichyl pyrophosphate Man9GlcNAc2 alpha-1,3-glucosyltransferase (*ALG6*) were used for further analysis of synteny comparison flanking TPC homologs among select insect genomes.

### In vitro cytosolic calcium measurements using Fura-2

SH-SY5Y cells and iPSC-derived midbrain dopaminergic neurons plated on coverslips were loaded with the ratiometric fluorescent Ca^2+^ indicator Fura-2 by incubation for 1 h at room temperature (RT) with the acetoxymethyl ester form of Fura-2 (Fura-2 AM, 2.5 µM) and 0.005% (vol/vol) pluronic acid (both from Invitrogen) in HEPES-buffered saline (HBS) containing 10 mM HEPES, 1.25 mM KH_2_PO4, 2 mM MgSO_4_, 3 mM KCl, 156 mM NaCl, 2 mM CaCl_2_, and 10 mM glucose (all from Sigma-Aldrich) at pH 7.4. S2R+ cells were loaded similarly except for the addition of 2.5 mM probenecid to prevent dye leakage. After labeling, cells were quickly washed three times in HBS and mounted in an imaging chamber (Bioscience Tools) with 1 ml HBS.

Epifluorescence images were captured every 3 s with a cooled coupled device camera (IMAGO; TILL Photonics) attached to an Olympus IX71 inverted fluorescence microscope that was fitted with a UApo/340 20×/0.70 W ∞/0.17 objective and a monochromatic light source (Polychrome IV) under the control of TILLvisION v4 software. The Fura-2 ratio was recorded using dual-excitation wavelengths of 340 and 380 nm, and a 440-nm long-pass filter was used to collect emitted fluorescence. Cells expressing GFP-tagged constructs were identified by excitation at 488 nm and capturing emitted fluorescence using a 515-nm long-pass filter. Cells expressing mRFP-tagged constructs were identified by excitation at 568 nm and capturing emitted fluorescence using a 590-nm long-pass filter. For some experiments, epifluorescence images were captured using a Megapixel monochrome cooled coupled device camera attached to an Olympus IX73 inverted fluorescence microscope that was fitted with a UPlanXApo 20×/0.80 ∞/0.17/OFN26.5 objective and a CoolLED multiple wavelength LED source under the control of MetaFluor 7.10.3.279 software. Fura-2 was excited at 340/380 nm, and emitted fluorescence was captured using a 425-nm long-pass filter.

Automated measurement of cytosolic Ca^2+^ in SH-SY5Y cells in a 96-well plate was performed using a fluorescence plate reader (CLARIOstar, BMG Labtech) under the control of Mars 3.42 R3 software. A single measurement comprised 16 flashes at 335 and 380 nm (each at 8-nm band-pass [BP]) while recording fluorescence at 520 nm (90-nm BP). Measurements were repeated on an individual well at 3-s intervals with 15 wells being recorded in parallel using “plate mode.” Defined volumes of HBS and modified HBS containing 120 mM KCl in place of NaCl were injected simultaneously through two independent injector needles to achieve final concentrations in the range of 10–90 mM KCl. Background fluorescence was measured from wells containing cells that were incubated with HBS without Fura-2.

### In vitro cytosolic Ca^2+^ measurements using genetically encoded Ca^2+^ indicators

SH-SY5Y cells plated on coverslips that transiently expressed GECO1.2 were mounted in an imaging chamber (Bioscience Tools) with 1 ml HBS and epifluorescence images captured every 3 s with a cooled coupled device camera (IMAGO; TILL Photonics) attached to an Olympus IX71 inverted fluorescence microscope that was fitted with a UApo/340 40×/1.35 Oil Iris ∞/0.17 objective and a monochromatic light source (Polychrome IV) under the control of TILLvisION v4 software. GECO1.2 was excited at 470 nm, and emitted fluorescence was collected using a 515-nm long-pass filter.

S2R+ cells plated on coverslips that transiently expressed GCaMP6s-tagged TPC2 constructs were mounted similarly and epifluorescence images captured every 3 s with a Megapixel monochrome cooled coupled device camera attached to an Olympus IX73 inverted fluorescence microscope that was fitted with a UPlanXApo 20×/0.80 ∞/0.17/OFN26.5 objective and a Cairn CoolLED multiple wavelength LED source under the control of MetaFluor 7.10.3.279 software. GCaMP6s was excited at 470 nm, and emitted fluorescence was collected using a 510-nm long-pass filter.

### Cell stimulations

In some experiments, cells were treated overnight with either 0.1% (vol/vol) DMSO (Sigma-Aldrich) or 1 µM vacuolin-1 (Chem Cruz) before being loaded with Fura-2. In other experiments, 1 mM LLOMe (Cayman Chemical Company) was included during loading. Either 0.1/0.2% (vol/vol) DMSO (Sigma-Aldrich) or H_2_O was used as controls when appropriate. SH-SY5Y cells were depolarized by replacing HBS with high-potassium buffer, containing 10 mM HEPES, 1.25 mM KH_2_PO4, 2 mM MgSO4, 156 mM KCl, 3 mM NaCl, 2 mM CaCl_2_, and 10 mM glucose (all from Sigma-Aldrich) at pH 7.4 such that the final KCl concentration was 50 mM. Cells were also acutely challenged with ionomycin, thapsigargin, PF-543, tetrandrine, ML-SI3 (synthesized as described previously [Leser et al., 2021]), riluzole and the TPC2 agonists, TPC2-A1-N, TPC2-A1-P, and SGA-10 (all synthesized as described previously [Gerndt et al., 2020]). Some stimulations were performed in nominally Ca^2+^-free HBS where Ca^2+^ was omitted from HBS.

### Imaging of lysosomes

To monitor lysosome integrity and morphology, SH-SY5Y cells plated on coverslips were loaded with 0.1 mg/ml dextran-conjugated rhodamine B (MW 10,000; Invitrogen) overnight at 37°C in culture. Cells were subsequently washed in dextran-free complete medium and cultured for a further 4 h (chase period) to label lysosomes. Epifluorescence and transmitted light images were captured with a cooled coupled device camera (IMAGO; TILL Photonics) attached to an Olympus IX71 inverted fluorescence microscope that was fitted with a UApo/340 20×/0.70 W ∞/0.17 objective and a monochromatic light source (Polychrome IV) under the control of TILLvisION v4 software. Rhodamine B was excited at 568 nm, and emitted fluorescence was captured with a 590-nm filter.

### Subcellular localization

Transfected SH-SY5Y and S2R^+^ cells plated on coverslips were fixed for 15 min with 4% (vol/vol) paraformaldehyde (VWR) prepared in phosphate-buffered saline (PBS; Sigma-Aldrich) at RT. After three 5-min washes in PBS, nuclei were labeled by incubation for 5 min with 1 µg/ml 4′,6-diamidino-2-phenylindole (DAPI; Sigma-Aldrich) at RT. Coverslips were washed thrice for 5 min with PBS, mounted onto microscope slides with 1,4-diazabicyclo[2,2,2]octane (Sigma-Aldrich), and sealed with colorless transparent nail varnish.

In some experiments, SH-SY5Y cells were stained for endogenous LAMP1 (Saito et al., 2023). Cells were fixed with paraformaldehyde, permeabilized with β-escin, blocked with bovine serum albumin/FBS, and sequentially incubated with antibodies and DAPI with intervening washes with Tween-20 prior to mounting using Fluoromount-G. Antibodies used were mouse LAMP1 antibody (H4A3) (1:10 dilution; catalog no. sc-20011 from Santa Cruz Biotechnology) and donkey anti-mouse (Alexa Fluor 594; 1:100 dilution; catalog no. A21203 from Thermo Fisher Scientific). In other experiments, S2R+ cells were incubated for 2 h at 37°C with 0.01% BioTracker 490 Green Cytoplasmic Membrane Dye (Merck) after fixation.

Confocal images of SH-SY5Y cells were taken using an inverted Axio Observer.Z1 microscope attached to a VivaTome scanner (Zeiss) fitted with an LCI Plan-Neofluar 63×/1.3 Imm Corr DIC M27 objective. GECO and GFP were excited using a 432- to 482-nm BP filter, and emitted fluorescence was captured using a 500- to 550-nm BP filter.

Confocal images of SH-SY5Y cells subject to immunocytochemistry, and S2R+ cells were acquired using an inverted Axio Observer.Z1 microscope attached to a LSM 880 confocal scanner (Zeiss) fitted with a Plan-Apochromat 63×/1.4 Oil DIC objective. DAPI, GFP/BioTracker 490 Green, and mCherry fluorescence were excited using wavelengths of 405, 488, and 561 nm, respectively. Emitted fluorescence was captured using either 410–479-nm, 490–577-nm, or 578–696-nm BP filters, respectively. 16-bit images were taken at 4× optical zoom.

All confocal images were acquired using ZEN Black software and processed on Fiji (ImageJ) software. For colocalization analyses, negative controls comprised the same image, but with the red channel rotated 180° to the right. Pearson’s correlation coefficient was obtained from confocal images using the “Just Another Co-localization Plugin” (JACoP).

### *Drosophila* husbandry and stocks

*Drosophila* were raised on standard cornmeal–yeast–sucrose medium at 25°C on a 12-h light:dark cycle.

UAS-TPC2-mCherry was created in pUAST-AttB (Bischof et al., 2007) and injected into the AttP2 site (RRID:BDSC_25710) at the NCBS *Drosophila* Facility (Bangalore). All other TPC2 constructs were injected by the *Drosophila* Injection Service at FlyORF (Zurich). *TH*-GAL4 (Friggi-Grelin et al., 2003) was a kind gift from Serge Birman (ESPCI, Paris, France), UAS-Lrrk2 and UAS-Lrrk2-G2019S (Liu et al., 2008) were kind gifts from Wanli Smith (Johns Hopkins, Baltimore, MD, USA), UAS-Lrrk2 R1114C and UAS-Lrrk2-G2019S/K1906M (Lin et al., 2010) were kind gifts from Cheng-Ting Chien (Academia Sinica, Taipei, Taiwan), UAS-Lamp1-GFP (Pulipparacharuvil et al., 2005) was a kind gift from Helmut Kramer (UT Southwestern, Dallas, TX, USA), and UAS-jGCaMP8m (Zhang et al., 2023) was a kind gift from James Jepson (UCL, London, UK). As a control, *TH*-GAL4 flies were crossed to the AttP2 landing site stock (empty vector), Bloomington Stocks Centre (RRID:BDSC_36303).

### SSVEP measurements

On the day of emergence, flies were placed in the dark or in disco-chambers at 29°C. 1-wk-old flies were prepared for SSVEP using a pooter and nail polish to secure them in a cutoff pipette tip, without anesthesia. Each fly was presented five times with a set of nine flickering stimuli. In each stimulus, the average light intensity was the same, but the amplitude of the flicker was adjusted from 10% to 100%, giving a range of contrasts. Fast Fourier transform (FFT) was applied to the responses, to separate the first harmonic (1F1), due to the photoreceptors, from the second harmonic (2F1), due to the lamina neurons. Stimuli were generated and responses recorded by an Arduino Due system with FFTs and contrast sensitivity computed in MATLAB. https://github.com/wadelab/flyCode

### Proboscis extension reflex measurements

Behavior was recorded from 1- to 7-day-old flies kept in the dark at 29°C. Flies were collected under CO_2_ anesthesia, and restrained by sticking them ventral side up to card with rubber cement (Fixo Gum). Flies were left to recover for 3 h at 29°C. They were presented with a droplet of 100 mM sucrose solution to the legs, and the immediate response (<2 s) was scored.

### Larval dissection and imaging

Third instar wandering larvae were immersed in PBS, on a Sylgard (DuPont) plate pinned at the anterior and posterior and the dorsal body wall cut along the midline from posterior to anterior. Fat bodies and internal organs were removed while leaving the CNS in place. PBS was then removed and replaced with 3.7% formaldehyde/PBS for 7 min. Preparations were then washed in PBT three times and then immersed in 70% glycerol/PBS for 2 h. Preparations were then mounted in Vectashield (Vectorlabs) and images collected from expressing dopaminergic neurons in the larval CNS on a Zeiss LSM 880 confocal microscope (inverted) with 10×/0.3 air or 63×/1.4 oil objectives. GFP was excited using 488-nm laser line and emission collected at 490–550 nm. mCherry was excited using 561-nm laser line and emission collected at 570–650 nm.

### Larval crawling measurements

Larval crawling assays were performed at 25°C using third instar wandering larvae. Two to three larvae were transferred onto the center of a 90-mm-diameter petri dish containing a thin layer of 1% agar and left to acclimatize for 1–2 min. The petri dish was placed upon a black surface and imaged from above using an iPhone 11 recording at 30 frames per second (fps) for 60 s. Videos were converted from .MOV to .MP4 format using HandBrake (https://handbrake.fr), with dimensions converted from 1080 × 1920 to 720 × 1280 pixels and 5 fps. Recordings were then converted to .avi format using ffmpeg (https://ffmpeg.org). Videos were then imported and analyzed in ImageJ using the MTrack2 plugin. Images were converted to 8-bit images and grayscale and the background subtracted to observe the larvae as white objects on a black background. Total track length was measured. For experiments testing the effects of TPC2 agonists, Formula 4-24 Instant Medium (Carolina Biological Supply) was supplemented with the drugs added from ethanol stocks.

### Ex vivo cytosolic Ca^2+^ measurements using jGCaMP8m

Intact third instar central nervous system (mouth hooks, eye-antennal disks, optic lobes, central brain, and ventral nerve cord) was quickly dissected in ice-cold sterile explant culture medium (∼4°C), consisting of Schneider’s *Drosophila* medium (Sigma-Aldrich) supplemented with 2.5 µg/ml human insulin (Sigma-Aldrich), 1% penicillin–streptomycin (Sigma-Aldrich), and 10% FBS (Sigma-Aldrich), under a dissecting microscope (Zeiss Stemi 2000). Explants were immobilized in an agarose scaffold as described previously (Bostock et al., 2020). Briefly, 1 ml 0.4% wt/vol low-temperature gelling agarose (Sigma-Aldrich) mixed with explant culture medium was heated to 42°C. The temperature of the agarose medium was then lowered to 34°C and pipetted into petri dishes (35 × 10 mm; Thermo Fisher Scientific) to which a single brain explant was added and oriented. The agarose was left to solidify for 15 min and the dish filled with sterile ice-cold explant culture medium comprising Schneider’s Insect medium (#S0146; Sigma-Aldrich) supplemented with 2.5 μl/ml human insulin (#I9278; Sigma-Aldrich), 1 % penicillin–streptomycin (#P4333; Sigma-Aldrich), and 10 % FBS (#F2442; Sigma-Aldrich).

Time-lapse Z-stacks were acquired using an upright Zeiss LSM 880 microscope with Spectra-Physics Mai Tai DeepSee two-photon lasers fitted with a W Plan-Apochromat 20×/1.0 DIC M27 70-mm objective. jGCaMP8m was excited at 935 nm (laser power <15%) and emission collected between 500 and 550 nm. 8-bit Z-stacks (10-40 slices per stack) were obtained with a Z interval of 1 µm and a scan time of 6–26 s per slice using ZEN Black software. Imaging was performed at 25°C.

Icy and ImageJ software were used to analyze movies. ROIs were manually drawn within every jGCaMP8m-expressing cell. The ROI intensity evolution plugin was used to extract the mean intensity evolution over time.

### Statistics

Data are presented as representative results from a single technical replicate (i.e., coverslip) or as the mean ± SEM from three or more independent experiments. Statistical analysis was performed using Prism 9 on datasets consisting of independent experiments performed on three or more different days per group/condition (i.e., *n* ≥ 3).

For two-group comparisons, an unpaired *t* test was used. For multiple groups, a one-way or two-way ANOVA followed by either Tukey’s, Sidak’s, or Dunnett’s tests was used. If the data were not normally distributed, we used a Mann–Whitney test (for two groups) or a Kruskal–Wallis H test followed by Dunn’s test (for multiple groups). The difference between comparisons was considered to be significant when P < 0.05. P values are indicated graphically: *P < 0.05, **P < 0.01, ***P < 0.001, ****P < 0.0001.

### Online supplemental material

Fig. S1 shows validation of LRRK2-expressing SH-SY5Y cell lines and effects of ROC/COR and kinase-inactivating mutations on cytosolic Ca^2+^. Fig. S2 shows effects of disrupting lysosomes on cytosolic Ca^2+^ in SH-SY5Y cells. Fig. S3 shows effects of TPC2 agonists on cytosolic Ca^2+^ in SH-SY5Y cells. Fig. S4 shows effects of TPC2 agonists on cytosolic Ca^2+^ in human dopaminergic neurons. Fig. S5 shows effects of TPC2 and LRRK2 expression in *Drosophila* dopaminergic neurons. Table S1 lists iPSC lines used in this study. Table S2 lists primers used for quantitative PCR. Table S3 lists primers used for cloning and mutagenesis. Video 1 shows Ca^2+^ measurements in the *Drosophila* brain.

## Supplementary Material

Table S1 lists iPSC lines used in this study.

Table S2 lists primers used for quantitative PCR.

Table S3 lists primers used for cloning and mutagenesis.

SourceData FS1 is the source file for Fig. S1.

## Data Availability

The data are available from the corresponding author upon reasonable request.
