# Supplementary material for: Lysosomal TPC2 channels disrupt Ca2+ entry and dopaminergic function in models of LRRK2-Parkinson’s disease
Source: J Cell Biol. 2025 Apr 25;224(6):e202412055. doi: 10.1083/jcb.202412055 (PMC12029513; doi:10.1083/jcb.202412055)
Supplement: Table S1 — lists iPSC lines used in this study. [file jcb_202412055_tables1.docx]

**Table S1. iPSC lines used in this study.**

| **PPM1 Cell line** | **Phenotype** | **Genotype** | **Gender** | **Age at biopsy** |
| --- | --- | --- | --- | --- |
| 3452 | Healthy | WT/WT | F | 64 |
| 3658 | Healthy | WT/WT | M | 58 |
| 51971 | PD | WT/G2019S | M | 64 |
| 51440 | PD | WT/G2019S | M | 67 |
| 40273 | PD | WT/G2019S | M | 61 |
