# Supplementary material for: Lysosomal TPC2 channels disrupt Ca2+ entry and dopaminergic function in models of LRRK2-Parkinson’s disease
Source: J Cell Biol. 2025 Apr 25;224(6):e202412055. doi: 10.1083/jcb.202412055 (PMC12029513; doi:10.1083/jcb.202412055)
Supplement: Table S2 — lists primers used for quantitative PCR. [file jcb_202412055_tables2.docx]

**Table S2. Primers used for quantitative PCR.**

| **Gene** | **Forward Primer** | **Reverse Primer (3’-5’)** |
| --- | --- | --- |
| LRRK2 | GGGATTTTGCAGGTCGTGAG | TGCCAACGAGAATCACAGGG |
| UBC | GAAGATGGACGCACCCTGTC | CCTTGTCTTGGATCTTTGCCTT |
