# Supplementary material for: Lysosomal TPC2 channels disrupt Ca2+ entry and dopaminergic function in models of LRRK2-Parkinson’s disease
Source: J Cell Biol. 2025 Apr 25;224(6):e202412055. doi: 10.1083/jcb.202412055 (PMC12029513; doi:10.1083/jcb.202412055)
Supplement: Table S3 — lists primers used for cloning and mutagenesis. [file jcb_202412055_tables3.docx]

**Table S3. Primers used for cloning and mutagenesis.**

| **Construct** | **Forward primer** | **Reverse primer** |
| --- | --- | --- |
| TPC2-GCaMP | ATTCGAATTCGCCACCATGGCGGAACCCCAGGC | TAGTTCTAGACGCTCACTTCGCTGTCATC |
| TPC2^L265P^-GCaMP | CTGACTTCCCCCCTGGTGCTG | AGACTCAGGCAGGTTCTG |
| TPC1-mCherry | CACCGAATTCATGGCTGTGAGTTTGGATGAC | CCTTGCGGCCGCGCCTTGAATTTGAGGTAACGGTCTGGGAGCG |
