# Supplementary figures and images for: Lysosomal TPC2 channels disrupt Ca2+ entry and dopaminergic function in models of LRRK2-Parkinson’s disease
Source: J Cell Biol. 2025 Apr 25;224(6):e202412055. doi: 10.1083/jcb.202412055 (PMC12029513; doi:10.1083/jcb.202412055)

LRRK2

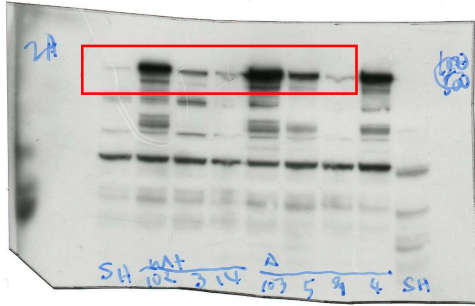

LRRK2

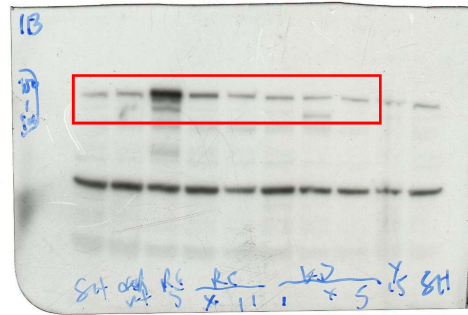

**a**

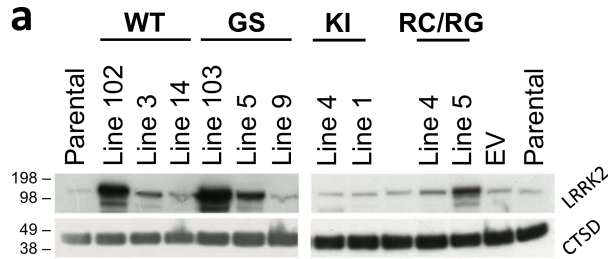

CTSD

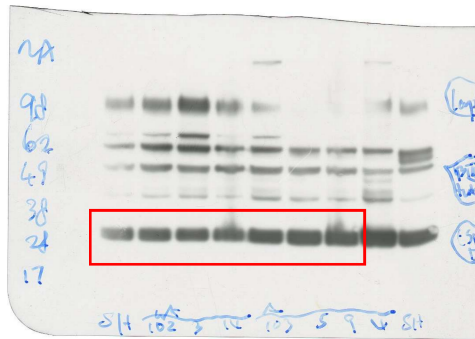

CTSD

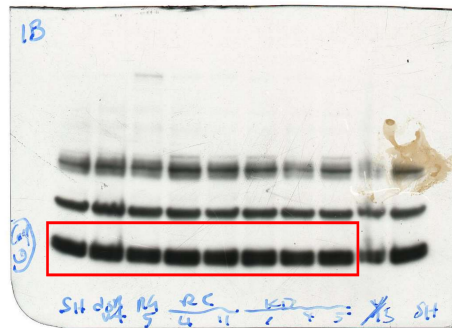

Supplement: SourceData FS1 — is the source file for Fig. S1. [file jcb_202412055_sourcedatafs1.pdf]
